# Isolation, Characterization, Complete Structural Assignment, and Anticancer Activities of the Methoxylated Flavonoids from *Rhamnus disperma* Roots

**DOI:** 10.3390/molecules26195827

**Published:** 2021-09-26

**Authors:** Hamdoon A. Mohammed, Mohammed F. Abd El-Wahab, Usama Shaheen, Abd El-Salam I. Mohammed, Ashraf N. Abdalla, Ehab A. Ragab

**Affiliations:** 1 Department of Medicinal Chemistry and Pharmacognosy, College of Pharmacy, Qassim University, Buraydah 51452, Saudi Arabia; 2 Department of Pharmacognosy, Faculty of Pharmacy, Al-Azhar University, Cairo 11371, Egypt; mohabdelwahab@yahoo.com (M.F.A.E.-W.); Usamayosef2003@yahoo.com (U.S.); ami07@fayoum.edu.eg (A.E.-S.I.M.); 3 Department of Pharmacology and Toxicology, Faculty of Pharmacy, Umm Al-Qura University, Makkah 21955, Saudi Arabia; ashraf_abdalla@hotmail.com; 4 Department of Pharmacology and Toxicology, National Center for Research, Khartoum 2404, Sudan

**Keywords:** *Rhamnus disperma*, flavonoids, methoxylated flavonoids, cytotoxicity, apoptosis, cell cycle

## Abstract

Different chromatographic methods including reversed-phase HPLC led to the isolation and purification of three *O*-methylated flavonoids; 5,4’-dihydroxy-3,6,7-tri-*O*-methyl flavone (penduletin) (**1**), 5,3’-dihydroxy-3,6,7,4’,5’-penta-*O*-methyl flavone (**2**), and 5-hydroxy-3,6,7,3’,4’,5’-hexa-*O*-methyl flavone (**3**) from *Rhamnus disperma* roots. Additionlly, four flavonoid glycosides; kampferol 7-*O*-*α*-L-rhamnopyranoside (**4**), isorhamnetin-3-*O*-*β*-D-glucopyranoside (**5**), quercetin 7-*O*-*α*-L-rhamnopyranoside (**6**), and kampferol 3, 7-di-*O*-*α*-L-rhamnopyranoside (**7**) along with benzyl-*O*-*β*-D-glucopyranoside (**8**) were successfully isolated. Complete structure characterization of these compounds was assigned based on NMR spectroscopic data, MS analyses, and comparison with the literature. The *O*-methyl protons and carbons of the three *O*-methylated flavonoids (**1**–**3**) were unambiguously assigned based on 2D NMR data. The occurrence of compounds **1**, **4**, **5**, and **8** in *Rhamnus disperma* is was reported here for the first time. Compound **3** was acetylated at 5-OH position to give 5-*O*-acetyl-3,6,7,3’,4’,5’-hexa-*O*-methyl flavone (**9**). Compound **1** exhibited the highest cytotoxic activity against MCF 7, A2780, and HT29 cancer cell lines with IC_50_ values at 2.17 µM, 0.53 µM, and 2.16 µM, respectively, and was 2–9 folds more selective against tested cancer cell lines compared to the normal human fetal lung fibroblasts (MRC5). It also doubled MCF 7 apoptotic populations and caused G_1_ cell cycle arrest. The acetylated compound **9** exhibited cytotoxic activity against MCF 7 and HT29 cancer cell lines with IC_50_ values at 2.19 µM and 3.18 µM, respectively, and was 6–8 folds more cytotoxic to tested cancer cell lines compared to the MRC5 cells.

## 1. Introduction

Rhamnus is a genus of about 110 species of shrubs or small- to medium-sized deciduous or evergreen trees, which are native from temperate to tropical regions and commonly known as buckthorns in the family Rhamnaceae [1,2]. *Rhamnus disperma* Ehrenb belongs to the family and is a native plant to the northern Middle East and Arabian Peninsula, including Saudi Arabia [3], Syria, Lebanon, Palestine, and considered one of Rhamnus species in the flora of Egypt [1,4]. The plant is reported to contain flavonoids, flavonoid glycosides, and phenolic constituents [5,6]. The Rhamnus species is reported for several biological activities including antioxidant, anti-acetylcholinesterase [7,8], anti-inflammatory, cytotoxic [9,10], and antimicrobial activities [11,12].

**Figure 1:**
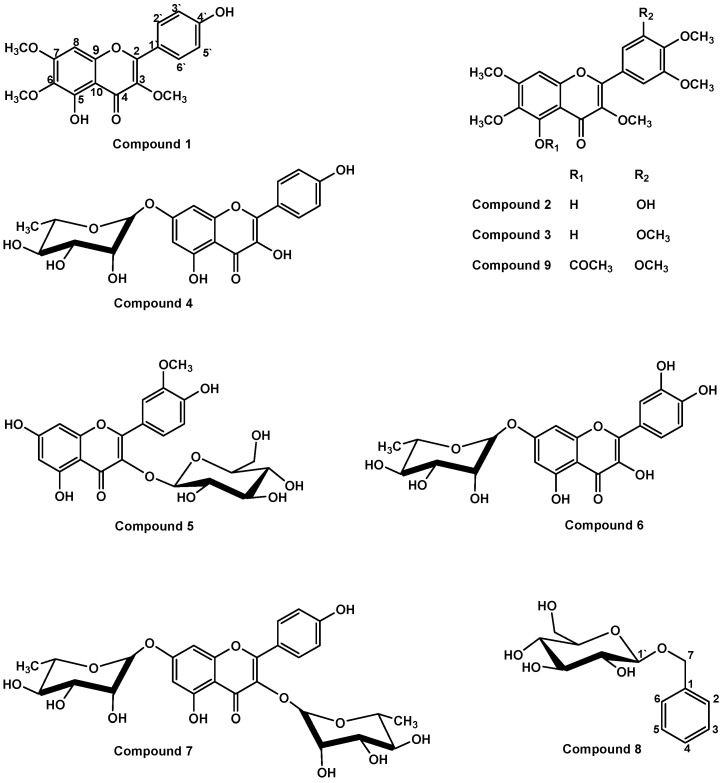
Chemical structures of compounds **1**–**9**.

The present study reported the isolation and structure characterization of three O-methylated flavonoids (compounds **1**–**3**), four flavonoid glycosides **(compounds 4**–**7**), and one benzyl glucoside (compound **8**) from the roots of *Rhamnus disperma* (Figure 1). The compounds’ cytotoxic activity against human breast adenocarcinoma (MCF 7), human ovary adenocarcinoma (A2780), human colon adenocarcinoma (HT 29) cell lines, and normal human fetal lung fibroblast (MRC5) was evaluated in the current work. The study also demonstrated the synthesis and characterization of the 5-*O*-acetylated product of compound **3** (compound **9**, Figure 1). The cytotoxic activity of compound **9** was also investigated against MCF 7, HT 29, and MRC5 cell lines as a part of the current study.

## 2. Results and Discussion

The ethyl acetate fraction of the alcoholic extract of *Rhamnus disperma* roots was repeatedly subjected to silica gel column chromatography (Si gel CC) to afford seven fractions (A-G). Fraction A was subjected to Si gel CC followed by HPLC analysis and separation yielding compounds **1**–**3**. Fractions F and G were subjected to SPE-C_18_ column followed by Si gel CC and sephadex LH-20 to give compounds **4**–**8** (Figure 1). The occurrence of compounds **1**, **4**, **5**, and **8** are reported in the current work for the first time from *Rhamnus disperma*. Although, compounds **2**, **3**, **6**, and **7** were previously isolated from the same plant [5,13]. The current work revised the chemical shifts of the *O*-methyl protons and carbons of the methoxyl groups of compounds **1**–**3**. The structures of the methoxylated flavonoids (**1**–**3**) were also confirmed through the unambiguous assignment based on 2D NMR data (spectra available in the Supplementary Materials). Furthermore, compound **3** was acetylated to give compound **9,** which was identified through 1D and 2D NMR analyses.

Compound (**1**) was obtained as yellow crystals and its ^1^H and ^13^C NMR data revealed that it is a methoxylated flavonoid. The ^1^H NMR spectrum of **1** showed three methoxyl groups which was evident from the three sharp protons signals at δ_H_ 3.92 (7-OMe), 3.80 (3-OMe), and 3.74 (6-OMe), which correlated in the HSQC spectrum to the corresponding methoxyl carbons at δ_C_ 57.0, 60.2, and 60.5, respectively. The location of the methoxy groups in **1** was deduced from the following HMBC correlations: 3H signal at δ_H_ 3.80 with C-3 (δ_C_ 138.1), 3H signal at δ_H_ 3.74 with C-6 (δ_C_ 132.1), and 3H signal at δ_H_ 3.92 with C-7 (δ_C_ 159.1). The downfield shift of C-2 (δ_C_ 156.5) and C-3 (δ_C_ 138.1) confirmed the position of the C-3 methoxylated aromatic carbon. Furthermore, the HMBC correlations between H-8 (δ_H_ 6.89) and C-6 (δ_C_ 132.1), C-7 (δ_C_ 159.1), C-9 (δ_C_ 152.3), and C-10 (δ_C_ 106.1) confirmed the position of the C-6 and C-7 methoxylated aromatic carbons.

Compound (**2**) was obtained as yellow crystals and its ^1^H and ^13^C NMR data revealed that it is also a methoxylated flavonoid. The ^1^H NMR spectrum of **2** showed five methoxyl protons at δ_H_ 3.90 (3H, s, 7-OMe), 3.85 (3H, s, 5’-OMe), 3.80 (3H, s, 3-OMe), 3.77 (3H, s, 4’-OMe), and 3.72 (3H, s, 6-OMe), which correlated in the HSQC spectrum to the corresponding methoxyl carbons at δ_C_ 56.9, 56.5, 60.4, 60.6, and 60.6, respectively. The location of the methoxyl groups in **2** was deduced from the following HMBC correlations: 3H signal at δ_H_ 3.80 with C-3 (δ_C_ 138.9), 3H signal at δ_H_ 3.72 with C-6 (δ_C_ 132.1), 3H signal at δ_H_ 3.90 with C-7 (δ_C_ 159.3), 3H signal at δ_H_ 3.77 with C-4’ (δ_C_ 139.5), and 3H signal at δ_H_ 3.85 with C-5’ (δ_C_ 153.5). The position of the methoxylated aromatic carbons at C-3, C-6, and C-7 was established as in compound **1** from the downfield shift of C-2 (δ_C_ 155.8) and C-3 (δ_C_ 138.9), and from the HMBC correlations between H-8 (δ_H_ 6.83) and C-6 (δ_C_ 132.1), C-7 (δ_C_ 159.3), C-9 (δ_C_ 152.3), and C-10 (δ_C_ 106.1). However, the HMBC correlation between H- 5 hydroxyl proton at δ_H_ 12.49 and C-5 (δ_C_ 152.0), C-6 (δ_C_ 132.1), and C-10 (δ_C_ 106.1) established the position of C-6 methoxylated aromatic carbon. Furthermore, the position of the methoxylated aromatic carbons at C-4’ and C-5’ was evident from the following HMBC correlations: H-2’ signal at δ_H_ 7.27 with C-2 (δ_C_ 155.8), C-1’ (δ_C_ 125.4), C-3’ (δ_C_ 151.0), C-4’ (δ_C_ 139.5), and C-6’ (δ_C_ 104.4); and the correlation between the H-6’ signal at δ_H_ 7.19 with C-2 (δ_C_ 155.8), C-1’ (δ_C_ 125.4), C-3’ (δ_C_ 151.0) C-4’ (δ_C_ 139.5), and C-5’ (δ_C_ 153.5).

Compound (**3**) was obtained as pale-yellow amorphous powder. The NMR data of **3** indicated that, it is a methoxylated flavonoid similar to that of **2** except the presence of an additional methyl group in **3**. Its ^1^H NMR spectrum of **3** showed six methoxyl protons at δ_H_ 3.84 (3H, s, 3-OMe), 3.73 (3H, s, 6-OMe), 3.92 (3H, s, 7-OMe), 3.88 (6H, s, 3’, 5’-OMe), and 3.77 (3H, s, 4’-OMe) which correlated in the HSQC spectrum to the corresponding methoxyl carbons at δ_C_ 60.4, 60.5, 57.0, 56.9, and 60.7, respectively. The location of the methoxyl groups in **3** was deduced from the following HMBC correlations: 3Hsignal at δ_H_ 3.84 with C-3 (δ_C_ 139.0), 3H signal at δ_H_ 3.73 with C-6 (δ_C_ 132.1), 3H signal at δ_H_ 3.92 with C-7 (δ_C_ 159.3), 3H signal at δ_H_ 3.77 with C-4’ (δ_C_ 140.5), and 6H signal at δ_H_ 3.88 with C-3’/5’ (δ_C_ 153.3). In a similar pattern to compound **2**, the position of the methoxylated aromatic carbons at C-3, C-6, and C-7 was established from the downfield shift of C-2 (δ_C_ 156.0) and C-3 (δ_C_ 139.0), and from the HMBC correlations between H-5 hydroxyl proton at δ_H_ 12.51 and C-5 (δ_C_ 152.0), C-6 (δ_C_ 132.1), and C-10 (δ_C_ 106.2); and the correlation between H-8 (δ_H_ 6.93) and the C-6 (δ_C_ 132.1), C-7 (δ_C_ 159.3), C-9 (δ_C_ 152.3), and C-10 (δ_C_ 106.2). Furthermore, the position of the methoxylated aromatic carbons at C-3’/C-4’ and C-5’ was evident from the following HMBC correlations: H-2’/H-6’ signal at δ_H_ 7.39 with C-2 (δ_C_ 156.0), C-1’ (δ_C_ 125.5), and C-2’ or C-6’ (δ_C_ 106.4), C-3’ or C-5’ (δ_C_ 153.3), and C-4’ (δ_C_ 140.5).

Compound (**4**) was obtained as yellow amorphous powder. The ^1^H NMR spectrum of compound **4**, showed two *meta*-coupled proton signals at δ 6.41 (1H, *J* = 1.8 Hz) and 6.82 (1H, *J* = 1.8 Hz), corresponding to H-6 and H-8 of the A-ring protons, respectively. A typical AA’BB’ system at δ 8.10 ppm (2H, d, *J* = 8.5 Hz, H-2’, H-6’) and δ 6.93 (2H, d, *J* = 8.8 Hz, H-3’, H-5’) confirmed the 1,4-disubstituted B-ring. These data in addition to the ion peak at *m/z* 286 in EIMS, indicated that the aglycon of **4** is kampferol [14]. The anomeric proton signal at δ_H_ 5.54 (brs, H-1”), in addition to the one doublet at δ_H_ 1.11 (*J* = 6.6 Hz, H-6”) observed in the ^1^H NMR spectrum of compound **4** established the presence of one rhamnose unit. The anomeric configuration for the rhamnose moiety was established to be in the *α*-configuration from its chemical shift and *^3^J*_H1,H2_ coupling constant [15]. The downfield shifts of H-6 and H-8 of the aglycone compared to those of H-6 and H-8 of kampferol indicates that rhamnose moiety was attached to the C-7 position [16,17,18]. Therefore, compound **4** was established as kampferol 7-*O*-*α*-L-rhamnopyranoside and in good agreement with the reported literature [19].

Compound (**5**) was obtained as yellow amorphous powder and was identified as isorhamnetin-3-*O*-*β*-D-glucopyranoside based on comparison of its NMR (^1^H and ^13^C) and EIMS data with those of the literature [17].

Compound (**6**) was obtained as yellow amorphous powder and its ^1^H NMR spectrum was similar to that of compound **4**, except for B-ring protons which appear as an ABX system in **6** [(δ_H_ 7.72 (1H, d, *J* = 2.0 Hz, H-2’), 7.59 (1H, dd, *J* = 8.3, 1.5 Hz, H-6’), and 6.89 (1H, d, *J* = 8.7 Hz, H-5’)] instead of AA’BB’ system in **4**. This indicated the presence of 1,3,4-trisubstituted B-ring in compound **6** versus 1,4-disubstituted B ring in compound **4**. These data indicated that the aglycone of **6** is a quercetin [14] which confirmed by the observed ion peak at *m/z* 302 in EIMS spectrum of **6**. The ^1^H NMR spectrum of **6** exhibited one anomeric proton signal at δ_H_ 5.55 (brs, H-1”) and the strong sharp doublet signal at δ_H_ 1.13 ppm (3H, d, *J* = 6.1 Hz, 6”-CH_3_) confirmed the rhamnose unit. From the above-mentioned data, compound **6** was established as quercetin-7-*O*-*α*-L-rhamnopyranoside and in good agreement with the reported literatures [19].

Compound (**7**) was obtained as yellow amorphous powder. The NMR data of the compound revealed the presence of dirhamnoside moieties attached to the kampferol aglycone. The compound was similar to compound **4** by having kampferol as an aglycone and one rhamnose unit at C-7 position in addition to another rhamnose unit at the C-3 position in compound **7**. Therefore, compound **7** was identified as kampferol 3, 7-di-*O*-*α*-L-rhamnopyranoside based on the comparison of its ^1^H and ^13^C NMR EIMS data with those of the literature [17].

Compound (**8**) was obtained as colorless oil and identified as benzyl-*O*-*β*-D-glucopyranoside based on comparison of its ^1^H, ^13^C NMR, and EIMS data with those of literature [19,20,21].

Compound (**9**) was obtained as pale-yellow amorphous powder. The ^1^H NMR spectrum of the acetylated compound **9** showed an aromatic singlet at δ_H_ 7.38 corresponding to H-8 proton of A ring and H-2’/H-6’ equivalent protons of B ring as indicated from HSQC correlation with C-8 (δ 99.6) and C-2’/C-6’ (δ_C_ 106.3). The six methoxyl groups were observed at δ_H_ 3.77 (s, 3-OMe), 3.74 (s, 6-OMe), 4.00 (s, 7-OMe), 3.89 (s, 3’, 5’-OMe), and 3.77 (s, 4’-OMe) which correlated to the corresponding methoxyl carbons at δ_C_ 60.2, 61.5, 57.3, 56.6, and 60.7, respectively, as showed in the HSQC spectrum. The observed ^1^H NMR singlet at δ_H_ 2.39 and ^13^C NMR signals at δ_C_ 21.2 and δ_C_ 169.4 indicated the presence of an acetyl group in **9**. The lack of the hydrogen-bonded C-5 hydroxyl group signal and the downfield shift of H-8 (δ_H_ 7.38) compared to H-8 (δ_H_ 6.93) of **3** together with the downfield shift of C-5 (153.0) and the upfield shift of C-4 carbonyl (172.6) compared to C-5 (152.0) and C-4 carbonyl (178.8) of **3** confirmed the acetylation of C-5 hydroxyl group in **9**. Therefore, the structure of **9** was identified as 5-*O*-acetyl-3,6,7,3’,4’,5’-hexa-*O*-methyl flavone.

### Cytotoxicity Assay

The result of MTT cytotoxicity assay of compounds **1**–**3** showed variable IC_50_ values against the three tested cancer cells ranging from 0.53 µM to 9.07 µM. However, other isolated compounds **4**–**7** did not show any inhibition of the cancerous cells proliferation at the tested concentrations. Among the three active compounds, compound **3** showed IC_50_ values of 2.76 ± 0.16, 3.73 ± 1.75, and 2.71 ± 1.25 µM against the MCF 7, A2780, and HT 29 cancer cell lines, respectively, and it was 4–5 folds more cytotoxic against cancer cell lines compared to the human fetal lung fibroblasts (MRC5) normal cells. Compound **2** was less active compared to compound **3**, as it exhibited IC_50_ value ranged from 6.81 µM to 9.07 µM against the three cancerous cell lines, and it was not selective for MRC5 normal cells as it showed IC_50_ value of 5.46 ± 1.57 µM against the normal MRC5 cells. Among the three methoxylated flavonoids, compound **1** showed the highest inhibition activity against both MCF 7 and HT 29 cells (IC_50_ value of ≤2 µM), and A2780 cells (IC_50_ value of 0.53 ± 0.45 µM). Importantly, compound **1** also showed 2–9 folds lower cytotoxicity against MRC5 cells with growth inhibition IC_50_ value of 4.40 ± 1.45 µM (Table 1). Following the process of acetylation, compound **9** was tested against MCF7 and HT29 cell lines. It showed IC_50_ values in the range of 2–3 µM. It is slightly more active than its precursor, compound **3**, against the MCF 7 cell line. Compound **9** was also 6–8 folds less cytotoxic to MRC5 cells compared to tested cancer cell lines (Table 1). The overall results showed for the higher cytotoxic activity of compounds **1**–**3** and **9** compared to the other flavonoids (compounds **4**–**7**) are mostly attributed to the presence of the methoxyl moities in the former group of compounds. This claim is completely supported by the literature which proves that poly methoxylated flavonoid derivatives are more potent as cytotoxic agents and have higher ability to inhibit the tumor cells than the flavonoid derivatives with free hydroxylated groups [22,23,24]. The literature also concluded that methoxy-flavonoids showed remarkable chemo-protective properties and are potentially useful as anticancer agents [25].

**Figure 2:**
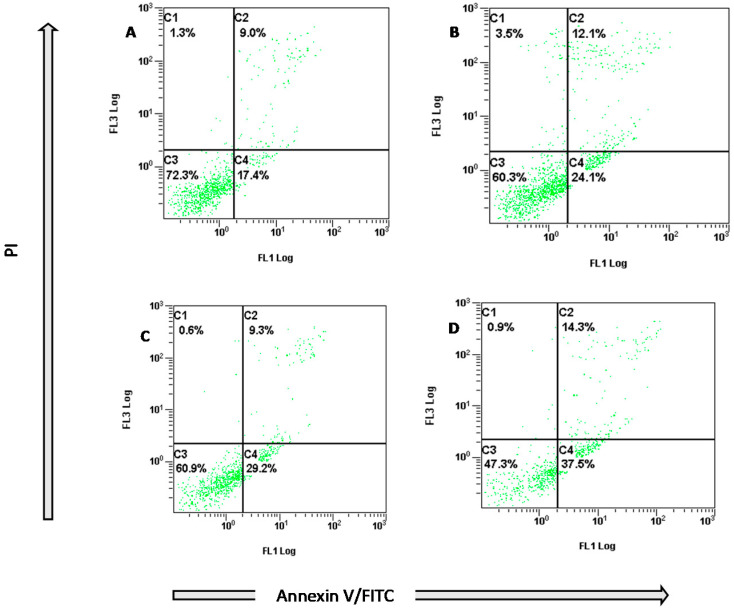
Histogram showing different phases of staining MCF7 cells with annexin V FITC/PI treated with compound **1** (24 h; A: 0, B: 5 µM, C: 10 µM and D: 20 µM). *X*-axis: annexin V, *Y*-axis: PI. C1: (necrosis-death, PI+/annexin V−); C2: (late apoptosis, PI+/annexin V+); C3: (living cells, PI−/annexin V−); C4: (early apoptosis, PI−/annexin V+). Experiment was repeated 3 × (*n* = 3).

Apoptosis was quantified by detecting cell surface exposure of phosphatidylserine (PS) in apoptotic cells using annexin V PI/FITC. Living cells stained with neither of the two dyes (PI−/annexin V−), while early apoptotic cells stain only with annexin V (PI−/annexin V+). In late apoptosis, cell membrane integrity is lost allowing penetration of PI (PI+/annexin V+); while in necrosis (death), cells stain with PI only (PI+/annexin V−). In this study, compound **1** induced remarkable apoptosis in a dose dependent manner (Figure 2).

**Figure 3:**
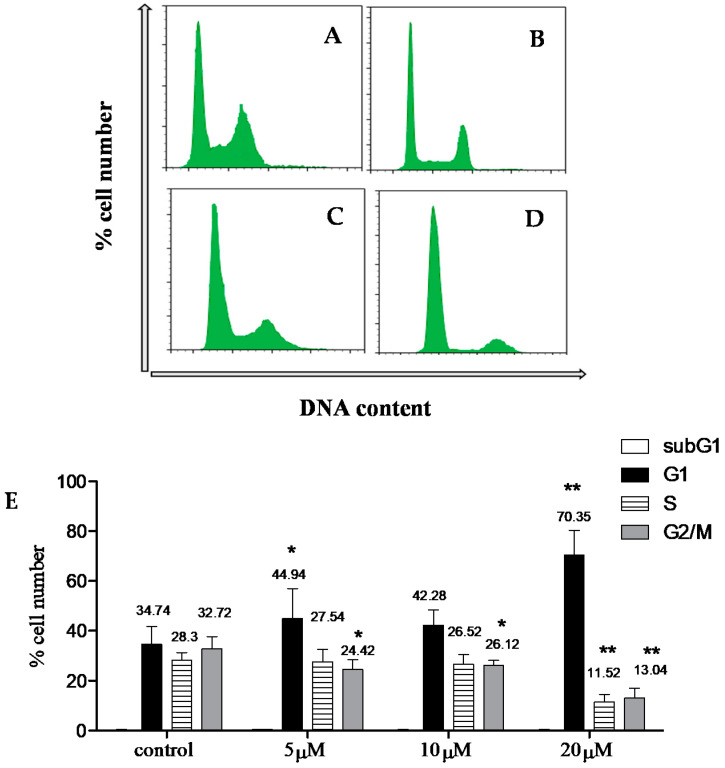
Graphs showing effect of compound 1 (24 h; (**A**): 0, (**B**): 5 µM, (**C**): 10 µM, and (**D**): 20 µM) in MCF 7 cell cycle distribution. (**E**): Data shown are mean % ± SD (*n* = 2). Experiment was repeated 3 × (*n* = 3). Statistical differences compared to untreated control cells were assessed by one-way ANOVA with the Tukey’s post-hoc multiple comparison test. *p* < 0.1 (*) and *p* < 0.01 (**) were taken as significant.

The cell cycle is a series of changes occurring from the initial phase of cell formation leading to its division as a consequence of a specific mechanism. Cell cycle phases include G_1_ (gap 1), S (synthesis), G_2_ (gap 2), and M (mitosis), with cell cycle arrest usually taking place in the G_1_/S or the G_2_/M check points. Compound **1** was used to treat the MCF 7 cell line (24 h; 5 µM, 10 µM, and 20 µM) to investigate possible cell cycle effect. Compared to the control, compound **1** caused dose-dependent G_1_ arrest (Figure 3).

## 3. Materials and Methods

### 3.1. General Experimental Procedure

UV spectra were collected with a Shimadzu UV-1650PC spectrophotometer; IR spectra were measured on a Shimadzu Infrared-400 spectrophotometer (Shimadzu, Kyoto, Japan). The ^1^H- and ^13^C- or ^13^C-APT NMR measurements were obtained with BrukerAvance III spectrometer operating at 500 or 400 MHz (for ^1^H) and 125 or 100 MHz (for ^13^C) in DMSO-*d_6_* solution, and chemical shifts were expressed in δ (ppm) with reference to TMS and coupling constant (*J*) in Hertz. ^13^C multiplicities were determined by the DEPT pulse sequence (135°) or APT experiment. HSQC and HMBC NMR experiments were carried out using Bruker AV-500 spectrometer. EIMS was carried on Scan EIMS-TIC, VG-ZAB-HF, X-mass (158.64, 800.00) mass spectrometer (VG Analytical, Inc., Palo Alto, CA, USA). Silica gel (Si gel 60, Merck, Kenilworth, NJ, USA) and sephadex LH-20 (Pharmacia, New Jersey, NJ, USA) were used for the open column chromatography. Solid phase extraction was performed on SPE-C_18_ cartridges (strata columns). Semi-prep HPLC was performed on Waters semi-prep HPLC (Waters, Milford, MA, USA), ODS column (Waters XBridge C-18, 5 μm, 10 × 150 mm Prep column); detector: PDA at 210–400 nm; flow rate: 2.0 mL/min, sample volume (loop): 100 mL. The entire system was controlled using Empower 3 Software. TLC was carried out on precoated silica gel 60 F254 (Merck) plates. Developed chromatograms were visualized by spraying with 1% vanillin-H_2_SO_4_, followed by heating at 100 °C for 5 min and by exposure to the vapors of a concentrated ammonia solution (25%).

### 3.2. Plant Material

The roots of *Rhamnus disperma* Ehrenb were collected from Saint Kathrin Protectorate, South Sinai, Egypt in April 2013, and were kindly identified by Dr. Ibraheem El-Garf, Professor of Plant Taxonomy, Faculty of Science, Cairo University, Egypt. A voucher specimen {RD2013} was deposited in the Pharmacognosy Department, Faculty of Pharmacy, Al-Azhar University, Cairo, Egypt. The collected roots were sliced into small pieces and subjected to shade drying at room temperature.

### 3.3. Extraction and Isolation

**Figure 4:**
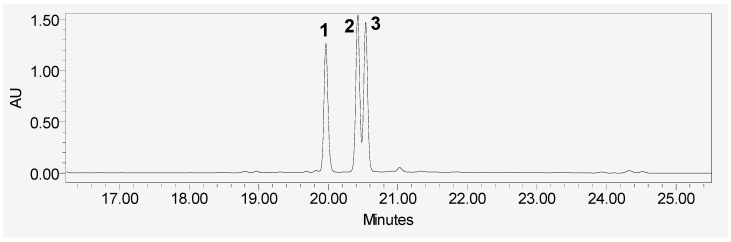
HPLC chromatogram of fraction A. Peaks 1, 2, and 3 referred to compound **1**, **2**, and **3**, respectively.

The air-dried powdered roots of *R. disperma* (700 g) were exhaustively extracted with ethyl alcohol (3 × 4 L). The total alcoholic extracts were combined and concentrated under vacuum to dryness at 40 °C which resulted in 145 g of the dried extract. The dried extract was suspended in 500 mL distilled water and defatted with petroleum ether. The defatted aqueous extract was partitioned with ethyl acetate (EtOAc) to give about 20 g of the ethyl acetate fraction. The remaining aqueous extract was partitioned with *n*-butanol to give about 40 g of the *n*-butanol fraction. The ethyl acetate fraction was applied to a column of Si gel and eluted with *n*-hexane-EtOAc (90:10→0:100) to give seven fractions of A to G. Fraction A (960 mg) was rechromatographed on a column of Si gel and eluted with chloroform to give four subfractions of A1 to A4. HPLC analysis of subfraction A3 (400 mg) showed three major peaks (Figure 4) which was separated by repeated semi-preparative HPLC on the ODS column. The solvent system used is a gradient starting at 30% methanol in 2% formic acid which was held for 5 min, followed by the gradual increase to 100% methanol after 25 min to give compound **1** (40 mg), compound **2** (30 mg) andcompound **3** (45 mg) with retention times 19.98, 20.41, and 20.53 min, respectively. Fraction F (950 mg) was subjected to Si gel column eluted with chloroform–methanol (100:0→80:20) to give three sub-fractions; F1 to F3. Subfraction F2 (300 mg) was rechromatographed over Si gel column using chloroform–methanol (100:0→90:10) to give two further subfractions of F2a and F2b. Subfraction F2a (90 mg) was purified by Sephadex LH-20 column (MeOH) to give compound **4** (8 mg). The subfraction F2b (110 mg) was subjected to Si gel C_18_ column chromatography eluted with water–methanol (100.0→30:70) to give compound **5** (10 mg) and compound **6** (11 mg). Fraction G (800 mg) was further applied to a column of Si gel and eluted with chloroform–methanol (95:5→70:30) to give two subfractions of G1 and G2. Subfraction G1 (390 mg) was subjected to an SPE-C_18_ cartridge eluted with H_2_O–CH_3_OH (100:0→50:50) to give three fractions of G1a, G1b and G1c. Fraction G1b (70 mg) was rechromatographed over Si gel column eluted with chloroform–methanol (90:10) followed by a column of Sephadex LH-20 (MeOH) to give compound **8** (12 mg). Subfraction G2 (220 mg) was subjected to an SPE-C_18_ cartridge eluted with H_2_O–CH_3_OH (20:10→50:50) to give two fractions of G2a and G2b. Fraction G2a (90 mg) was rechromatographed over Si gel column eluted with chloroform–methanol (80:20) followed by a column of Sephadex LH-20 (MeOH) to give compound **7** (18 mg) (the isolation scheme is provided in the Supplementary Materials).

### 3.4. Spectroscopic Analysis of the Isolated Compounds 

Spectra are provided in the Supplementary Materials.

**5,4’-dihydroxy-3,6,7-tri-*O*-methyl flavone** (**penduletin**) **(1):** Yellow crystals [MeOH]; UV λ_max_ (MeOH) nm: 257sh, 276, 340, IR υ_max_ (KBr) cm^−1^: 3380, 2923, 1660, 1610, 1463, 1193; ^1^H NMR (DMSO-*d_6_*, 500 MHz) δ 12.63 (1H, brs, 5-OH), 7.99 (1H, d, *J* = 7.5 Hz, H-2’, H-6’), 6.97 (1H, d, *J* = 7.5 Hz, H-3’, H-5’), 6.89 (s, H-8), 3.92 (3H, s, 7-OCH_3_), 3.80 (3H, s, 3-OCH_3_), 3.74 (3H, s, 6-OCH_3_); ^13^C NMR (DMSO-*d_6_*, 125 MHz) δ 178.7 (C-4), 160.9 (C-4’), 159.1 (C-7), 156.5 (C-2), 152.3 (C-9), 152.2 (C-5), 138.1 (C-3), 132.1 (C-6), 130.7 (C-2’, C-6’), 120.9 (C-1’), 116.2 (C-3’, C-5’), 106.1 (C-10), 91.9 (C-8), 60.5 (6-OCH_3_), 60.2 (3-OCH_3_), 57.0 (7-OCH_3_); EIMS *m/z* 344 [M]^+^, 343 [M-1]^+^, 329 [M-15]^+^.

**5,3’-dihydroxy-3,6,7,4’,5’-penta-*O*-methyl flavone (2):** Yellow crystals [MeOH]; UV λ_max_ (MeOH) nm: 255sh, 273, 334, IR υ_max_ (KBr) cm^−1^: 3385, 2920, 1655, 1615, 1462, 1190; ^1^H NMR (DMSO-*d_6_*, 500 MHz) δ 12.49 (1H, brs, 5-OH), 9.70 (1H, brs, 3’-OH), 7.27 (1H, s, H-2’), 7.19 (1H, s, H-6’), 6.83 (s, H-8), 3.90 (3H, s, 7-OCH_3_), 3.85 (3H, s, 5’-OCH_3_), 3.80 (3H, s, 3-OCH_3_), 3.77 (3H, s, 4’-OCH_3_), 3.72 (3H, s, 6-OCH_3_); ^13^C NMR (DMSO-*d_6_*, 125 MHz) δ 178.82 (C-4), 159.3 (C-7), 155.8 (C-2), 153.5 (C-5’), 152.3 (C-9), 152.0 (C-5), 151.0 (C-3’), 139.5 (C-4’), 138.9 (C-3), 132.1 (C-6), 125.4 (C-1’), 110.4 (C-2’), 106.1 (C-10), 104.4 (C-6’), 91.9 (C-8), 60.6 (6-OCH_3_ and 4’-OCH_3_), 60.4 (3-OCH_3_), 56.9 (7-OCH_3_), 56.5 (5’-OCH_3_); EIMS *m/z* 404 [M]^+^, 403 [M-1]^+^, 389 [M-15]^+^.

**5-hydroxy-3,6,7,3’,4’,5’-hexa-*O*-methyl flavone (3):** Pale-yellow amorphous powder [MeOH]; UV λ_max_ (MeOH) nm: 265sh, 274, 280sh, 334, IR υ_max_ (KBr) cm^−1^: 3388, 2923, 1652, 1613, 1462, 1192; ^1^H NMR (DMSO-*d_6_*, 500 MHz) δ 12.51 (1H, brs, 5-OH), 7.39 (2H, s, H-2’, H-6’), 6.93 (s, H-8), 3.92 (3H, s, 7-OCH_3_), 3.88 (6H, s, 3’-OCH_3_, 5’-OCH_3_), 3.84 (3H, s, 3-OCH_3_), 3.77 (3H, s, 4’-OCH_3_), 3.73 (3H, s, 6-OCH_3_); ^13^C NMR (DMSO-*d_6_*, 125 MHz) δ 178.8 (C-4), 159.3 (C-7), 156.0 (C-2), 153.3 (C-3’, C-5’), 152.0 (C-5), 152.3 (C-9), 140.5 (C-4’), 139.0 (C-3), 132.1 (C-6), 125.5 (C-1’), 106.4 (C-2’, C-6’), 106.2 (C-10), 92.1 (C-8), 60.7 (4’-OCH_3_), 60.5 (6-OCH_3_), 60.4 (3-OCH_3_), 57.0 (7-OCH_3_), 56.9 (3’, 5’-OCH_3_); EIMS *m/z* 418 [M]^+^, 417 [M-1]^+^, 403 [M-15]^+^.

**kampferol 7-*O*-*α*-L-rhamnopyranoside (4):** Yellow amorphous powder [MeOH]; UV λ_max_ (MeOH) nm: 246sh, 267, 315sh, 351; IR υ_max_ (KBr) cm^−1^: 3440, 1655, 1600, 1515; ^1^H NMR (DMSO-*d_6_*, 400 MHz) aglycone δ 8.10 (2H, d, *J* = 8.5 Hz, H-2’, H-6’), 6.93 (2H, d, *J* = 8.8 Hz, H-3’, H-5’), 6.82 (1H, d, *J* = 1.8 Hz, H-8), 6.41 (1H, d, *J* = 1.8 Hz, H-6); sugar moiety δ 5.54 (1H, s, H-1”), 3.66–3.29 (4H, m, H-2”-H-5”), 1.11 (3H, d, *J* = 6.6 Hz, H-6”); EIMS *m/z* 286 [M-rhamnosyl]^+^.

**Isorhamnetin 3-*O*-*β*-D-glucopyranoside(5):** Yellow amorphous powder (MeOH); UV λ_max_ (MeOH) nm: 254, 266sh, 355; IR υ_max_ (KBr) cm^−1^: 3365, 2925, 1660, 1615, 1490, 1190; ^1^H-NMR (DMSO-*d_6_*, 400 MHz) aglycone δ 12.65 (1H, s, OH-5), 7.94 (1H, s, H-2′), 7.50 (1H, d, *J* = 8.4 Hz, H-6′), 6.92 (1H, d, *J* = 8.3 Hz, H-5′), 6.43 (1H, s, H-8), 6.20(1H, s, H-6), 3.84 (3H, s, 3’-OCH_3_); sugar moiety δ 5.57 (1H, d, *J* = 7.2 Hz, H-1”), 3.65–3.12 (5H, m, H-2”-H_2_-6”); ^13^C-APT NMR (DMSO-*d_6_*, 100 MHz) aglycone δ177.8 (C, C-4), 165.0 (C, C-7), 161.7 (C, C-5), 156.9 (C, C-2), 156.7 (C, C-9), 149.9 (C, C-3’), 147.4 (C, C-4’), 133.4 (C, C-3), 122.5 (CH, C-6’), 121.6 (C, C-1’), 115.7 (CH, C-5’), 113.9 (C, C-2’), 104.4 (C, C-10), 99.3 (CH, C-6), 94.2 (CH, C-8), 56.1 (CH_3_, 3’-OCH_3_); sugar moiety δ 101.3 (CH, C-1”), 77.9 (CH, C-3”), 76.9 (CH, C-5”), 74.8 (CH, C-2”), 70.3 (CH, C-4”), 61.1 (CH_2_, C-6”); EIMS *m*/*z* 316 [M-glucosyl]^+^.

**Quercetin 7-*O*-*α*-L-rhamnopyranoside (6):** Yellow amorphous powder [MeOH]; UV λ_max_ (MeOH) nm: 255, 268sh, 374; IR υ_max_ (KBr) cm^−1^: 3450, 1655, 1650, 1590; ^1^H NMR (DMSO-*d_6_*, 400 MHz) aglycone δ 12.67 (1H, s, OH-5), 7.72 (1H, d, *J* = 2.0 Hz, H-2’), 7.59 (1H, dd, *J* = 8.3, 1.5 Hz, H-6’), 6.89 (1H, d, *J* = 8.7 Hz, H-5’), 6.79 (1H, d, *J* = 1.8 Hz, H-8), 6.41 (1H, d, *J* = 1.9 Hz, H-6), sugar moiety δ 5.55 (1H, s, H-1”), 3.66–3.12 (4H, m, H-2”-H-5”), 1.13 (3H, d, *J* = 6.1 Hz, H-6”); EIMS *m/z* 302 [M-rhamnosyl]^+^.

**Kampferol 3,7-di-*O*-*α*-L-rhamnopyranoside (7):** Yellow amorphous powder [MeOH]; UV λ_max_(MeOH) nm: 244sh, 266, 315sh, 350; IR υ_max_ (KBr) cm^−1^: 3445, 1660, 1610, 1515; ^1^H NMR (DMSO-*d_6_*, 400 MHz) aglycone δ 12.59 (1H, s, OH-5), 7.79(2H, d, *J* = 8.7 Hz, H-2’, H-6’), 6.92 (2H, d, *J* = 8.7 Hz, H-3’, H-5’), 6.78 (1H, d, *J* = 2.0 Hz, H-8), 6.45 (1H, d, *J* = 2.0 Hz, H-6); sugar moieties δ 5.55 (1H, d, *J* = 1.8 Hz, H-1”), 5.30 (1H, d, *J* = 1.4 Hz, H-1”’), 3.66–3.14 (8H, m, H-2”, H-2”’-H-5”, H-5”’), 1.14 (3H, d, *J* = 6.1 Hz, H-6”), 0.81 (3H, d, *J* = 5.5 Hz, H-6”’); ^13^C-APT NMR (DMSO-*d_6_*, 100 MHz) aglycone δ 178.4 (C, C-4), 162.2 (C, C-7), 161.4 (C, C-5), 160.6 (C, C-4’), 158.3 (C, C-2), 156.6 (C, C-9), 135.0 (C, C-3), 131.2 (CH, C-2’, 6’), 120.8, (C, C-1’), 115.9 (CH, C-3’, 5’), 106.2 (C, C-10), 99.9 (CH, C-6), 95.0 (CH, C-8); sugar moieties δ 102.3 (CH, C-1”), 98.9 (CH, C-1”’), 72.1, (CH, C-4”), 71.6 (CH, C-4”’), 71.1 (CH, C-2”), 70.8 (CH, C-2”’), 70.7 (CH, C-3”), 70.5 (CH, C-5”, C-3”’), 70.3 (CH, C-5”’), 18.4 (CH_3_, C-6”’), 17.9 (CH_3_, C-6”’); EIMS *m/z* 432 [M-one rhamnosyl]^+^ and 286 [M-two rhamnosyls]^+^.

**Benzyl-*O*-*β*-D-glucopyranoside (8):** Colorless oil; UV λ_max_ (MeOH) nm: 257, IRυ_max_ (KBr) cm^−1^: 3430 (OH), 2922 (CH), 1650, 1595 (aromatic); ^1^H NMR (DMSO-*d_6_*, 400 MHz) aglycone δ 7.40 (2H, d, *J*= 6.9 Hz, H-2, H-6), 7.34 (2H, t, *J*= 7.0 Hz, H-3, H-5), 7.29 (1H, m, H-4), 4.83 (1H, d, *J*= 12.2 Hz, H-7a), 4.58 (1H, d, *J*= 12.2 Hz, H-7b); sugar moiety δ 4.24 (1H, d, *J*= 7.6 Hz, H-1’), 3.71 (1H, d, *J*= 11.9 Hz, H-6’a), 3.47 (1H, m, H-6’b), 3.43–3.05 (4H, m, H-2’-H-5’); ^13^C NMR(DMSO-*d_6_*, 100 MHz) aglycone δ 138.5 (C, C-1), 128.6 (CH, C-3, C-5), 128.1 (CH, C-2, C-6), 127.8 (CH, C-4); sugar moiety δ 102.5 (CH, C-1’), 77.4 (CH, C-5’), 77.2 (CH, C-3’), 74.0 (CH, C-2’), 70.6 (CH, C-4’), 70.0 (CH_2_, C-7), 61.7 (CH_2_, C-6’); EIMS *m/z* 108 [M-glucosyl]^+^.

### 3.5. Acetylation of Compound **3**

Accurately, 30 mg of compound **3** was heated at 130 °C for 4 h with 2.0 mL of Ac,O according to the method described in Neves et al. [26]. The mixture was poured into ice-water, the precipitate was filtered off, and the acetylated compound was purified on silica gel CC with CH_2_Cl_2_–EtOAc (40:1) to give compound **9** (12 mg).

**5-*O*-acetyl-3,6,7,3’,4’,5’-hexa-*O*-methyl flavone** (**9**): Pale-yellow amorphous powder [MeOH]; ^1^H NMR (DMSO-*d_6_*, 500 MHz) δ 7.38 (3H, s, H-8, H-2’, H-6’), 4.00 (3H, s, 7-OCH_3_), 3.89 (6H, s, 3’-OCH_3_, 5’-OCH_3_), 3.77 (6H, s, 3-OCH_3_, 4’-OCH_3_), 3.74 (3H, s, 6-OCH_3_), acetyl δ 2.39 (3H, s, CH_3_); ^13^C NMR (DMSO-*d_6_*, 125 MHz) δ 172.6 (C-4), 157.9 (C-7), 153.7 (C-2), 153.7 (C-3’, C-5’), 153.0 (C-5), 140.2 (C-9), 141.2 (C-3), 140.8 (C-4’), 139.2 (C-6), 125.7 (C-1’), 106.3 (C-2’, C-6’), 111.1 (C-10), 99.6 (C-8), 61.5 (6-OCH_3_), 60.7 (4’-OCH_3_), 60.2 (3-OCH_3_) 57.3 (7-OCH_3_), 56.6 (3’, 5’-OCH_3_), acetyl δ 169.4 (CO), 21,2 (CH_3_); EIMS *m/z* 460 [M]^+^.

### 3.6. Acid Hydrolysis of Compounds **4–8**

5 mg of each compound was separately refluxed with 2M HCl in MeOH (5 mL) at 80 °C for 4 h in a water bath. The reaction mixture was evaporated, and the hydrolysate after dilution with H_2_O (10 mL) was extracted with CHCl_3_ (3 × 10 mL). The CHCl_3_ extracts were evaporated to afford the aglycones, which were identified as kampferol for **4** and **7**, isorhamnetin for **5**, quercetin for **6** and benzyl alcohol for **8** by comparison with authentic samples, respectively. The aqueous layer was neutralized with sodium carbonate and concentrated to 1 mL under reduced pressure. The residue was compared with standard sugars by Si gel TLC [(CHCl_3_-MeOH-H_2_O:30:12:4), 9 mL of lower layer and 1 mL of HOAc], which indicated the sugars to be L-rhamnose in compounds **4**, **6** and **7** and glucose in compounds **5** and **8**.

### 3.7. Cytotoxicity Assay

Three cancerous cell lines, as well as one normal fibroblast cell line, were bought from the ATCC, USA: MCF 7 (human breast adenocarcinoma), A2780 (human ovary adenocarcinoma), HT 29 (human colon adenocarcinoma), and MRC5 (normal human fetal lung fibroblast). The culture procedure was performed as described in the literature [27,28]. Briefly, the cells were sub-cultured at 37 °C, 5% CO_2_, 95% air, and 100% relative humidity. RPMI-1640 media (10% FBS, L-glutamine and 1% antibiotic-antimycotic) was used for all cells except for MRC5, which was maintained in Eagles minimum essential medium (EMEM, 10% FBS and 1% antibiotic-antimycotic). Cytotoxicity of compounds **1**–**7** and **9** was evaluated by an MTT assay as previously described [29]. Each of the three cell lines and one normal fibroblast were cultured in 96-well plates (3 × 10^3^/well), and incubated at 37 °C overnight. Final concentrations of each compound were: 0, 0.05, 0.5, 5, 25, and 50 μM in media (DMSO 0.1%). Each concentration was tested in triplicates. The plates were incubated for 72 h and then MTT (50 μL) was added to each well. Plates were incubated for 3 h, supernatant was aspirated, and 100 μL of DMSO was added to each well. Absorbance was read on a multi-plate reader. The optical density of the purple formazan A550 is proportional to the number of viable cells. Compound concentrations causing 50% inhibition (IC_50_) compared to control cell growth (100%) were determined. GraphPad Prism version 5.00 for Windows, GraphPad Software (San Diego, CA, USA) was used for analysis.

### 3.8. Annexin VFITC/PI Apoptosis Assay

The Annexin V FITC/PI assay was devised to quantify apoptosis using MCF 7 cells [30]. MCF 7 cells were cultured in 6 well plates (1 × 10^5^ cells/well) in 2 mL medium overnight at 37 °C. Then it was treated by compound 1 (24 h; final concentrations: 0, 5, 10, and 20 µM). Media was collected in tubes and kept on ice, and remaining cells were trypsinized, incubated at 37 °C and added to previous tubes. Cells were centrifuged (350 g) and supernatants were discarded. Cells were washed with PBS, centrifuged and pellets re-suspended in binding buffer (100 μL) and annexin V FITC (10 μL). Tubes were incubated at room temperature in the dark for 20 min. Binding buffer (400 μL) and 10 μL propidium iodide (PI) were added. Samples were analyzed by flow cytometry (BC FC500) within 1 h. Viable cells were differentiated from early and late apoptotic/necrotic cells by annexin V (X axis) and PI staining (Y axis).

### 3.9. Cell Cycle Analysis

Cell cycle analysis was performed using MCF 7 cells, which were cultured in 6 well plates (1 × 10^5^ cells/well in 2 mL medium), before treatment with compound 1 (0, 5, 10, and 20 μM; 24 h). Cells were washed with cold PBS and trypsinized. Collected cells were centrifuged at 350 g/5 min, and supernatant discarded. Pellets were washed in cold PBS, centrifuged, and fixed overnight in 70% ice-cold ethanol. Centrifuged cells were re-suspended in cold PBS with the addition of ribonuclease A (15 min), followed by PI (2 µL/mL). Samples were held on ice and analyzed by flow cytometry. Data analysis of the DNA contents (PI bound to DNA) of 20,000 events was carried out using Expo 32 software [31].

### 3.10. Statistical Analysis

Samples were tested in triplicate, and each assay was repeated three times. Data were expressed as mean and standard deviation (mean ± SD). Comparisons were performed using GraphPad Prism version 5.00 for Windows.

## 4. Conclusions

In this study, eight compounds **1**–**8** were isolated from the roots of *Rhamnus disperma*. Compounds **1**, **4**, **5,** and **8** were isolated for the first time from the plant. Compound **1** exhibited the highest cytotoxic activity against three cancer cell lines: MCF 7, A2780, and HT 29, with IC_50_ values of 0.53–2.17 µM and was 2–9 folds more selective against tested cancer cell lines compared to the normal fibroblast cells (MRC5). It also doubled MCF 7 apoptotic populations in a dose dependent manner and caused G_1_ cell cycle arrest. It is concluded that compound **1** has cytotoxic, pro-apoptotic, and cell cycle arrest activities, and could have anticancer activity, which should be further tested using in vivo animal models.

## Figures and Tables

**Table 1:** Activity of the half-maximal inhibitory concentration (IC_50_ µM ± SD; 72 h) of compounds **1**–**3** and **9** against three cancer cell lines and one normal fibroblast cell line evaluated by MTT assay (72 h).

Compounds	MCF 7	A2780	HT 29	MRC5
**1**	2.17 ± 0.26	0.53 ± 0.45	2.16 ± 0.02	4.40 ± 1.45
**2**	6.81 ± 0.04	8.97 ± 1.72	9.07 ± 0.22	5.46 ± 1.57
**3**	2.76 ± 0.16	3.73 ± 1.75	2.71 ± 1.25	11.73 ± 1.58
**9**	2.190 ± 0.64	-	3.18 ± 0.64	19.04 ± 2.98

## Data Availability

All data are available in the manuscript and Supplementary File.

## Supplementary Materials

The following are available online, Figures S1–S31: NMR spectra of the compounds; Scheme S1: Extraction, fractionation and isolation of chemical constituents of *Rhamnus disperma* roots.

## Sample Availability

Samples of the compounds are not available.
