# Supplementary material for: Isolation, Characterization, Complete Structural Assignment, and Anticancer Activities of the Methoxylated Flavonoids from Rhamnus disperma Roots
Source: Molecules. 2021 Sep 26;26(19):5827. doi: 10.3390/molecules26195827 (PMC8510169; doi:10.3390/molecules26195827)
Supplement: Supplementary file 1 [file molecules-26-05827-s001.zip › molecules-1373314-supplementary.pdf]

Supplementary material

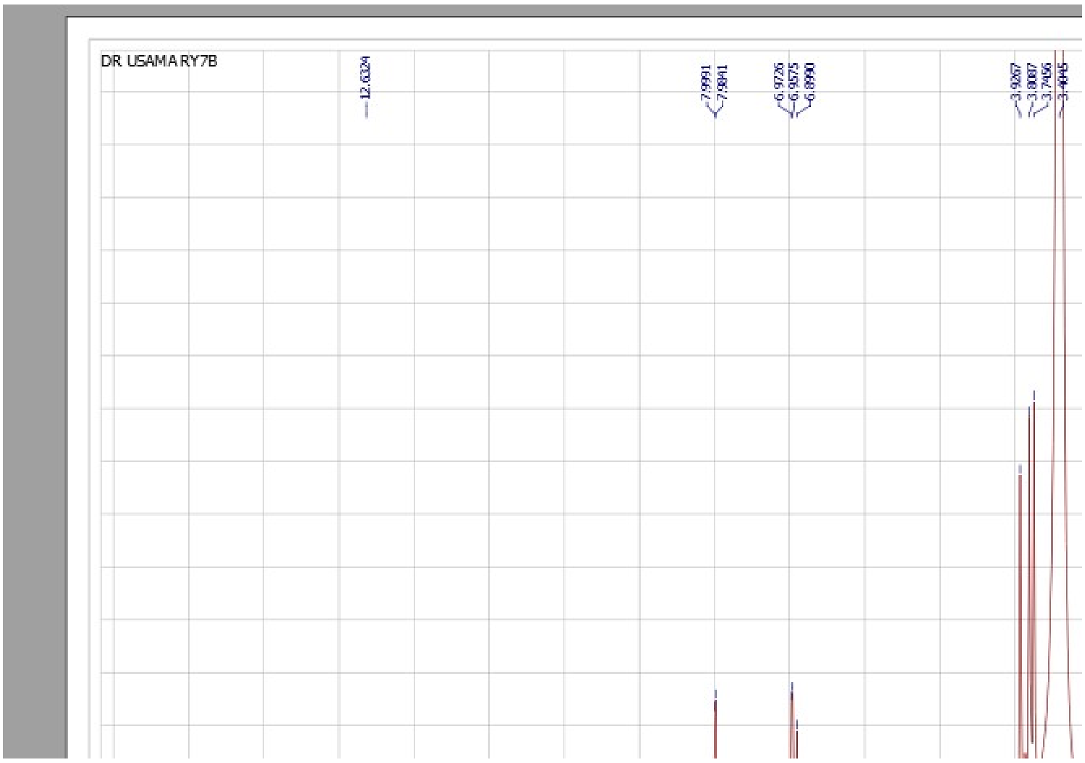

Figure S1: ^{1}H NMR spectrum of compound 1 (DMSO-d_6, 500 MHz)

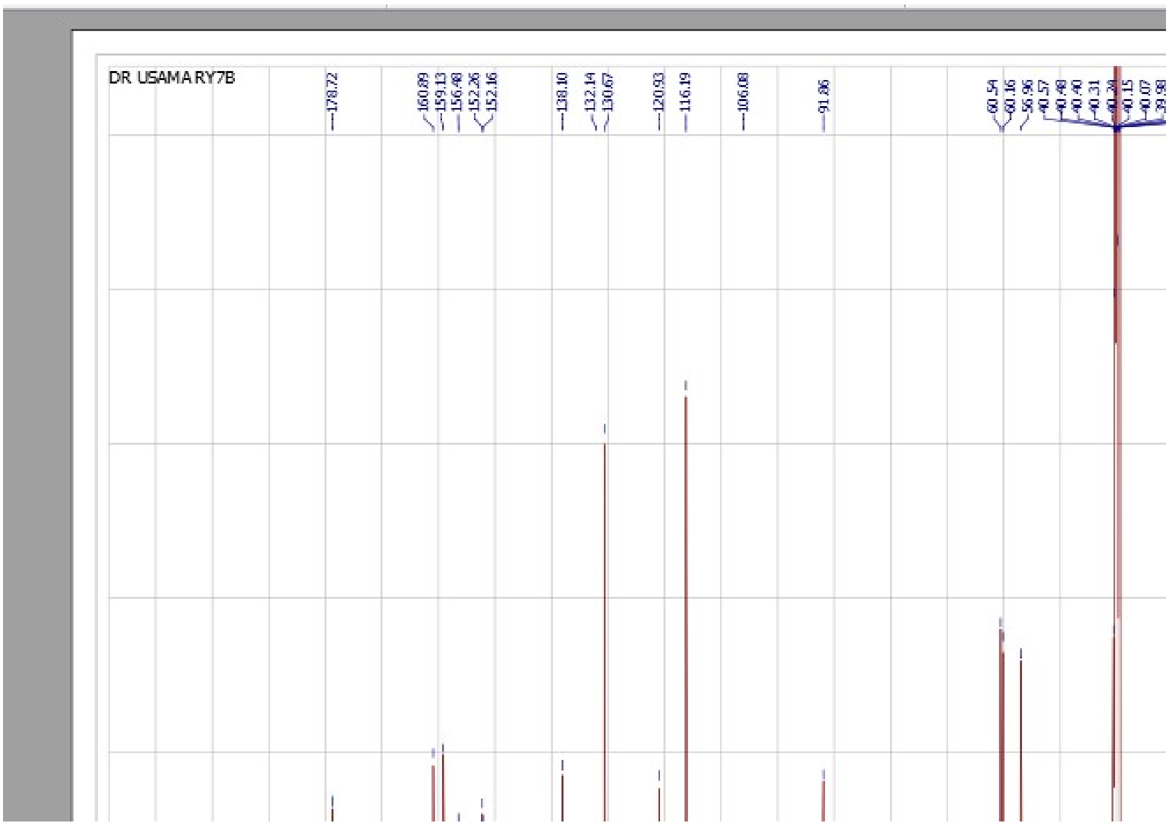

Figure S2: ^{13}C NMR spectrum of compound 1 (DMSO-d_6, 500 MHz)

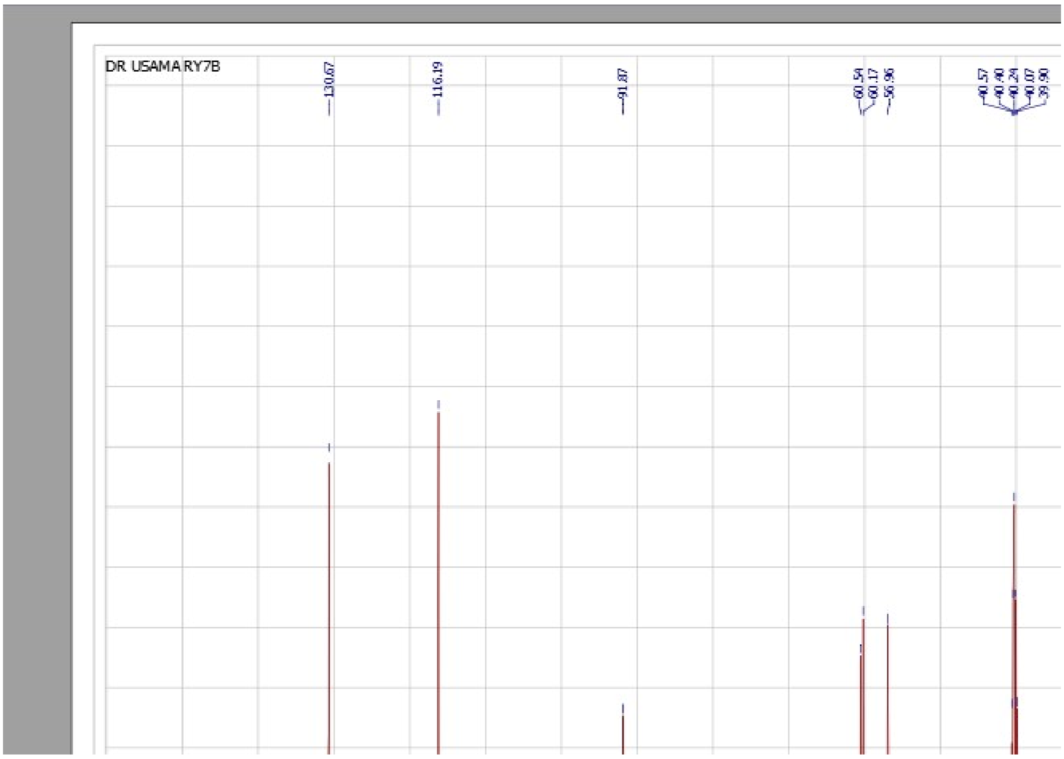

Figure S3: ^{13}C-DEPT NMR spectrum of compound 1 (DMSO-d_6, 500 MHz)

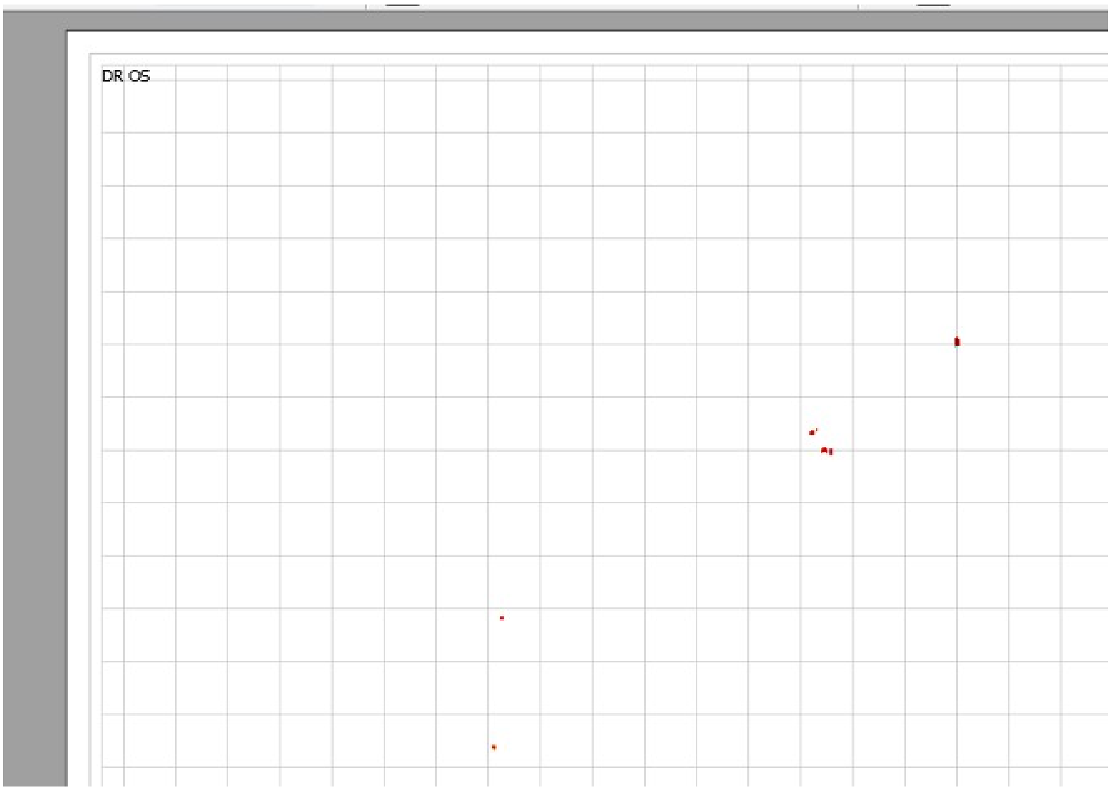

Figure S4: HSQC spectrum of compound 1 (DMSO-d_6, 500 MHz)

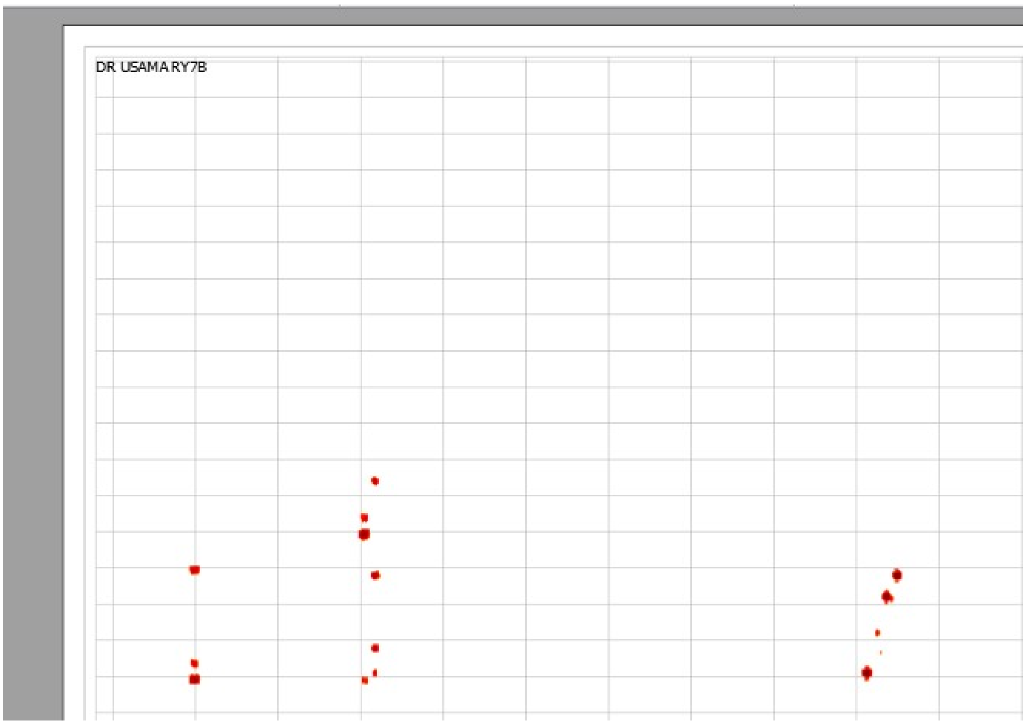

Figure S5: HMBC spectrum of compound 1 (DMSO-d_6, 500 MHz)

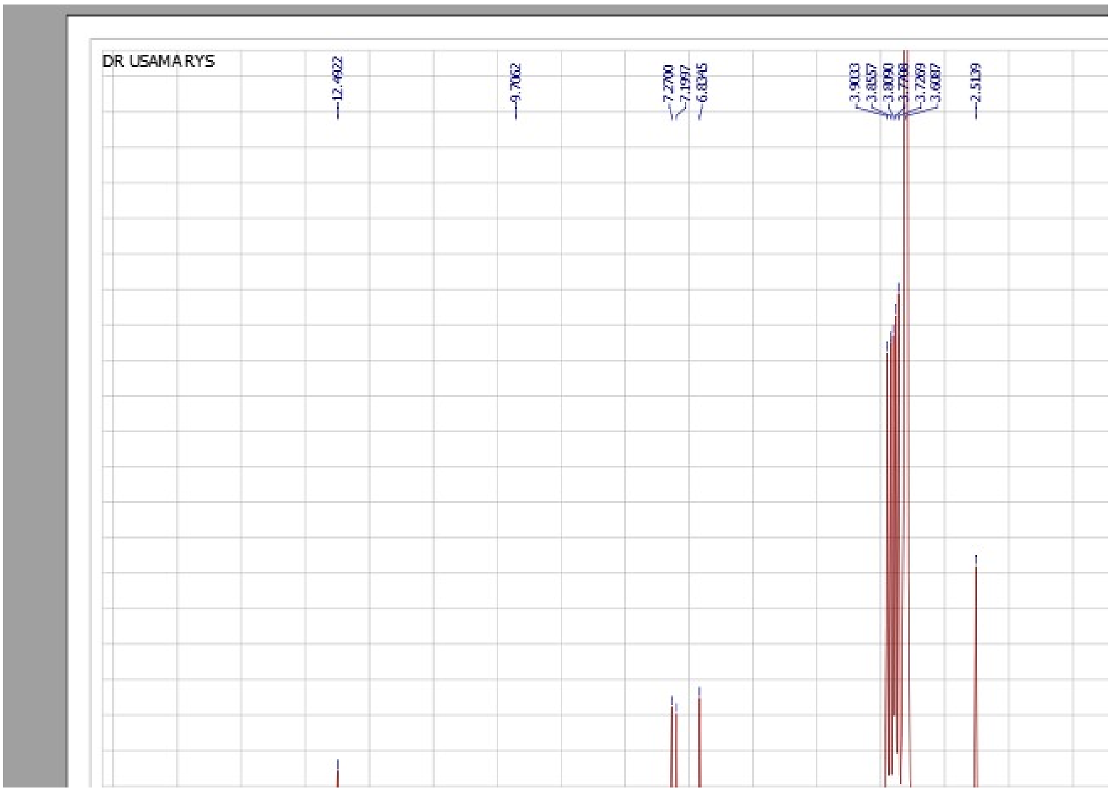

Figure S6: ^{1}H NMR spectrum of compound 2 (DMSO-d_6, 500 MHz)

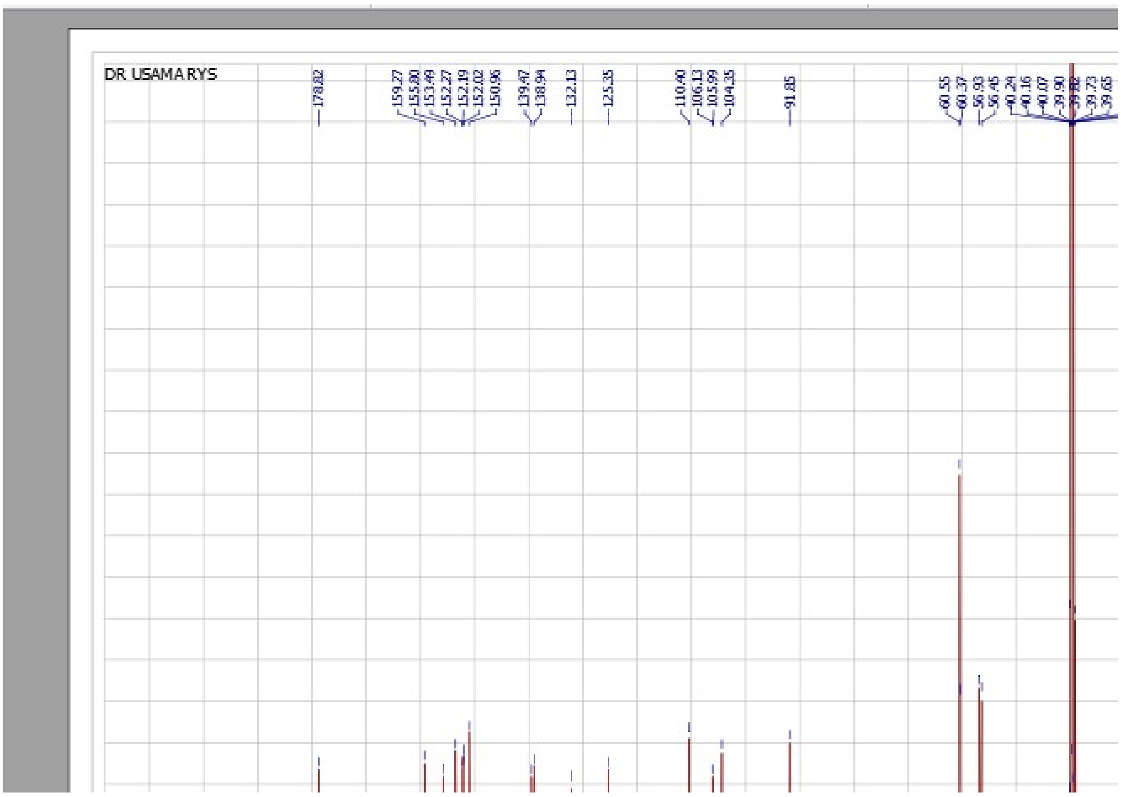

Figure S7: ^{13}C NMR spectrum of compound 2 (DMSO-d_6, 500 MHz)

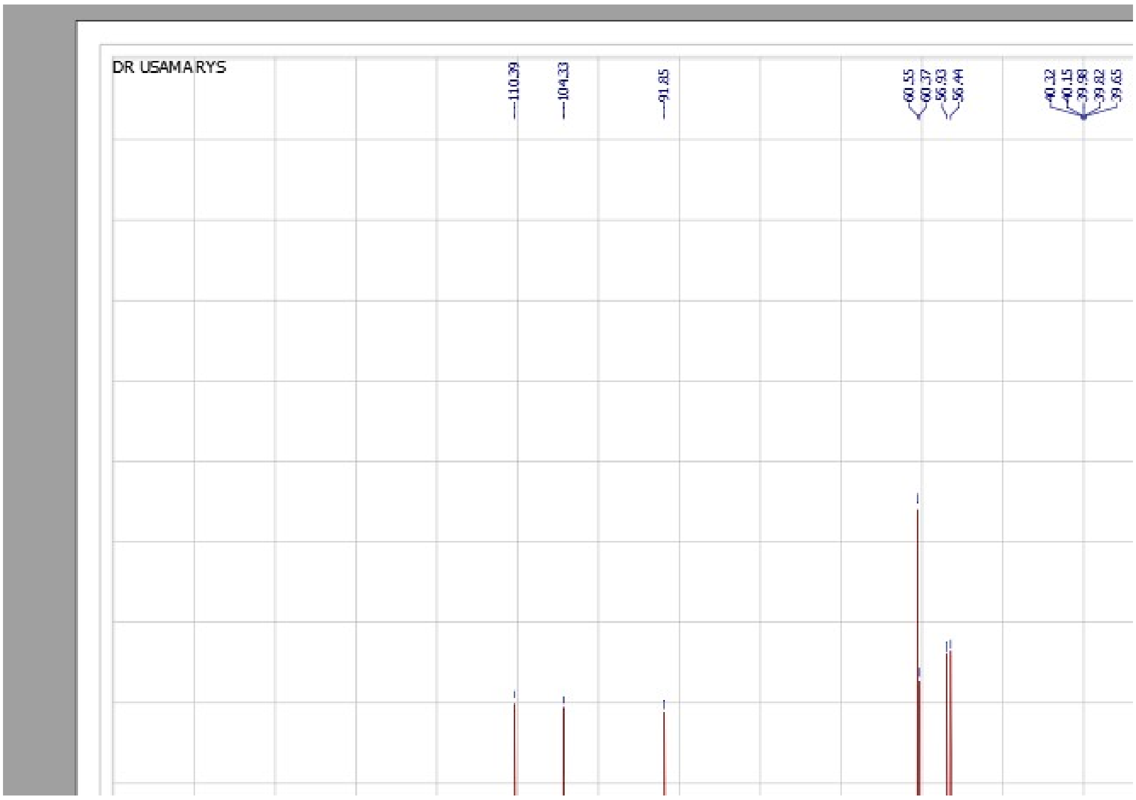

Figure S8: ^{13}C-DEPT NMR spectrum of compound 2 (DMSO-d_6, 500 MHz)

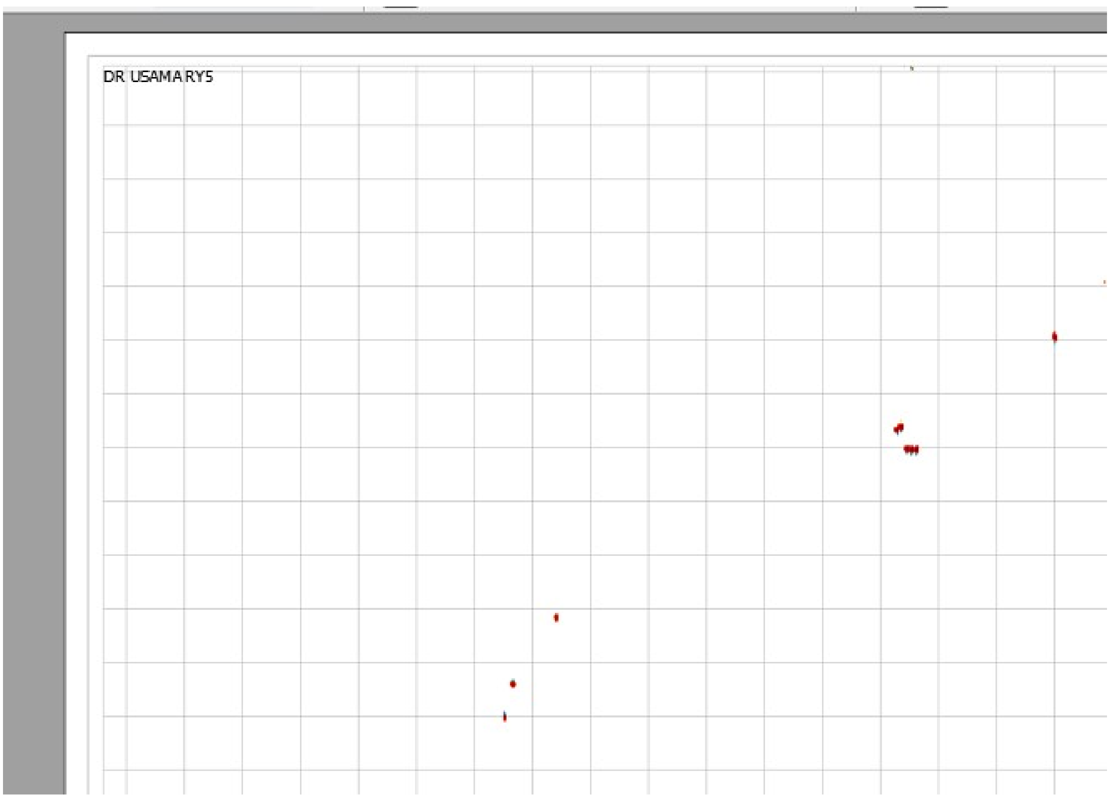

Figure S9: HSQC spectrum of compound 2 (DMSO-d_6, 500 MHz)

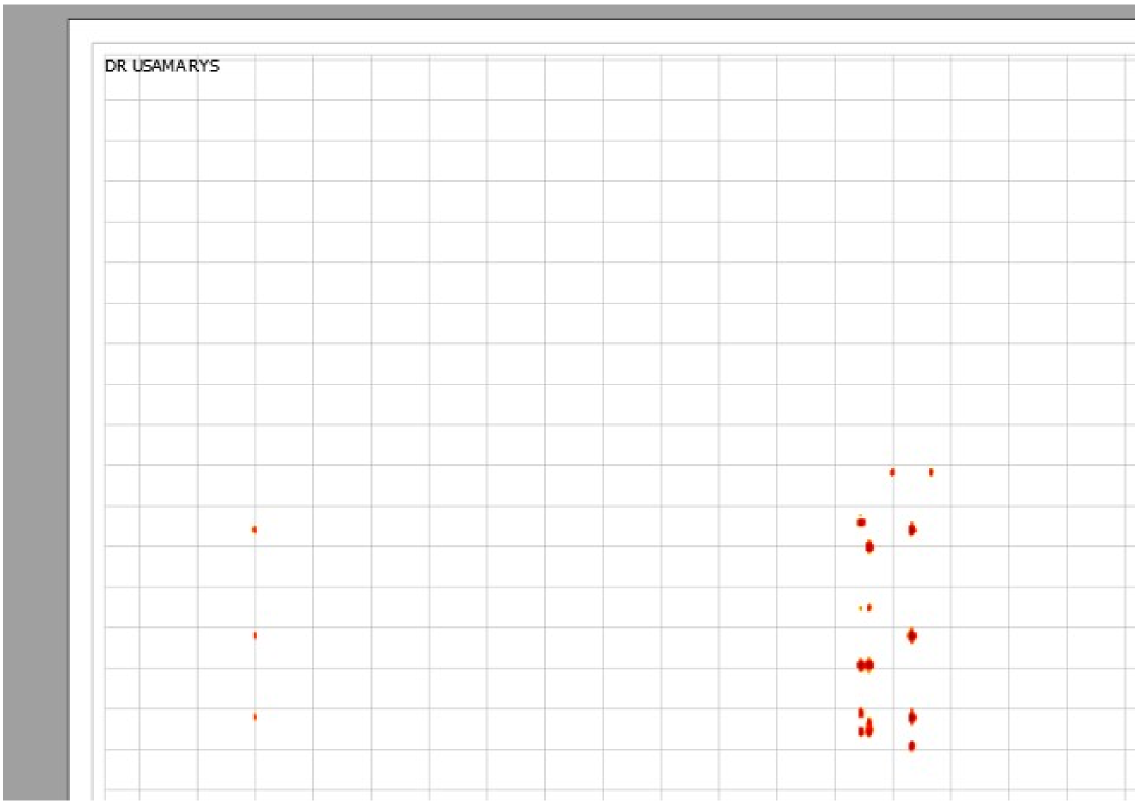

Figure S10: HMBC spectrum of compound 2 (DMSO-d_6, 500 MHz)

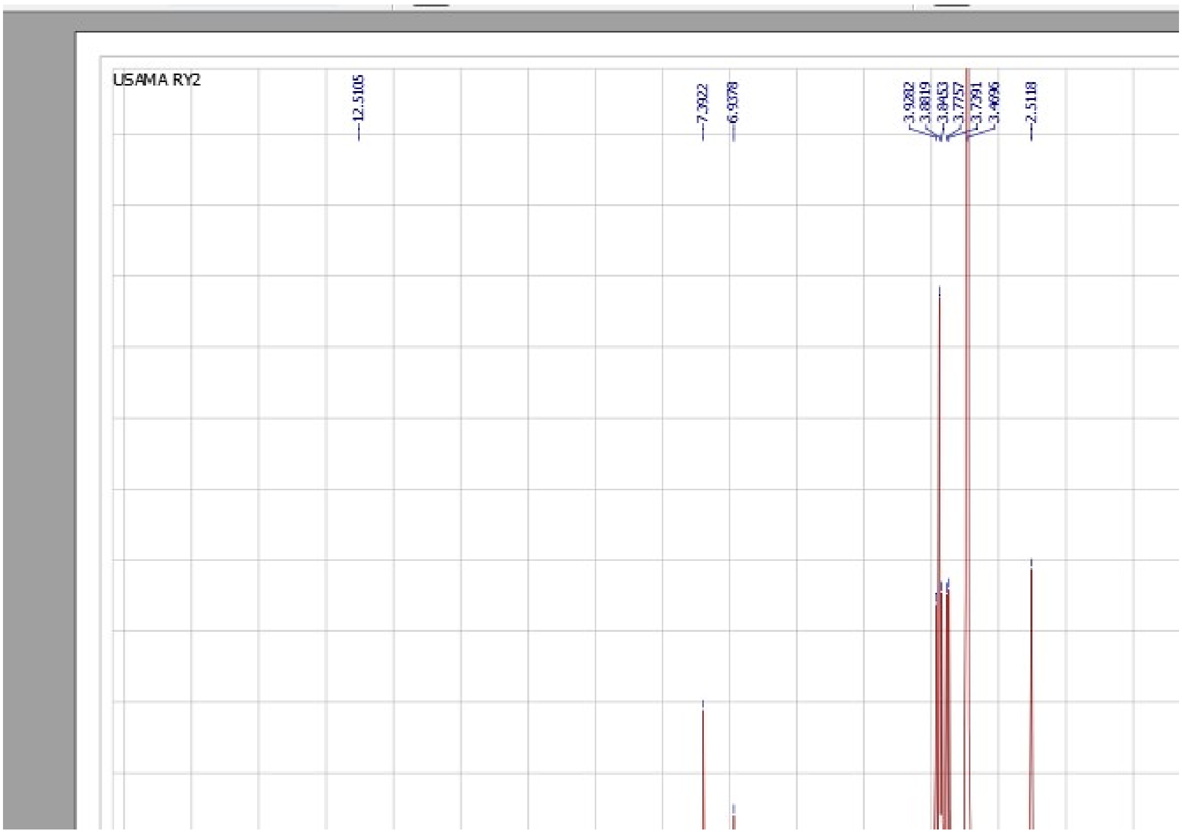

Figure S11: ^1H NMR spectrum of compound 3 (DMSO-d_6, 500 MHz)

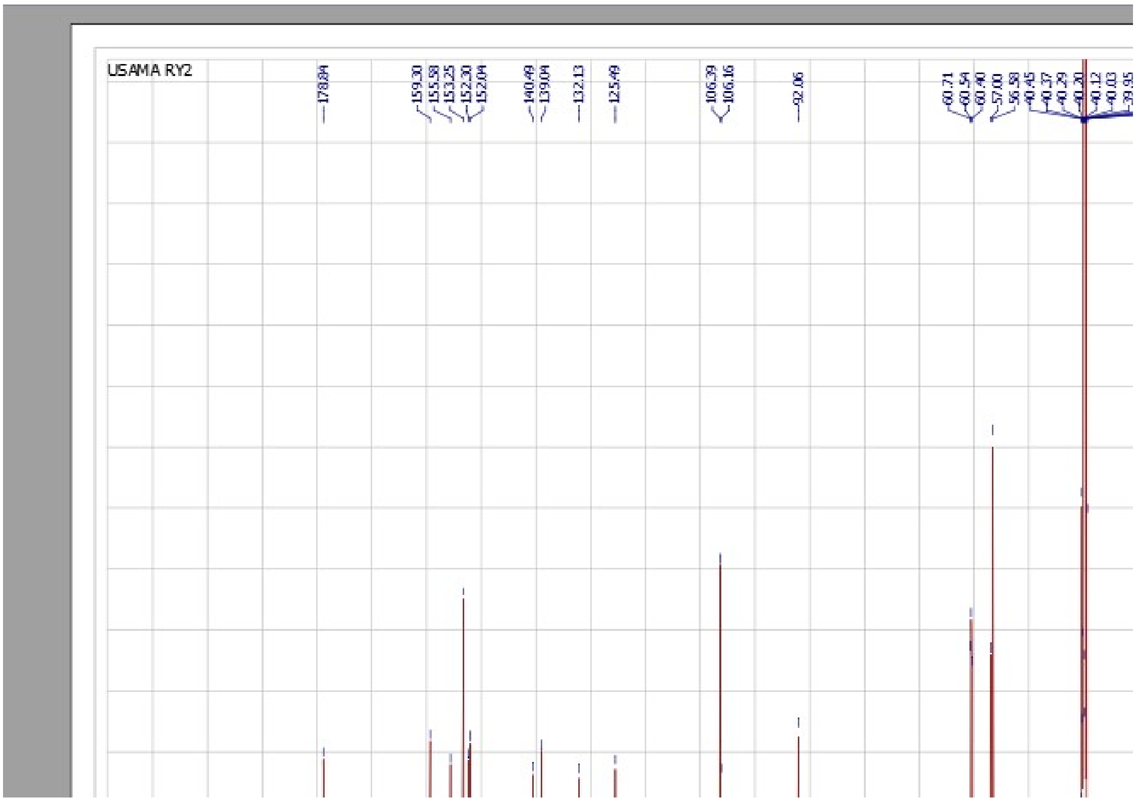

Figure S12: ^{13}C NMR spectrum of compound 3 (DMSO-d_6, 500 MHz)

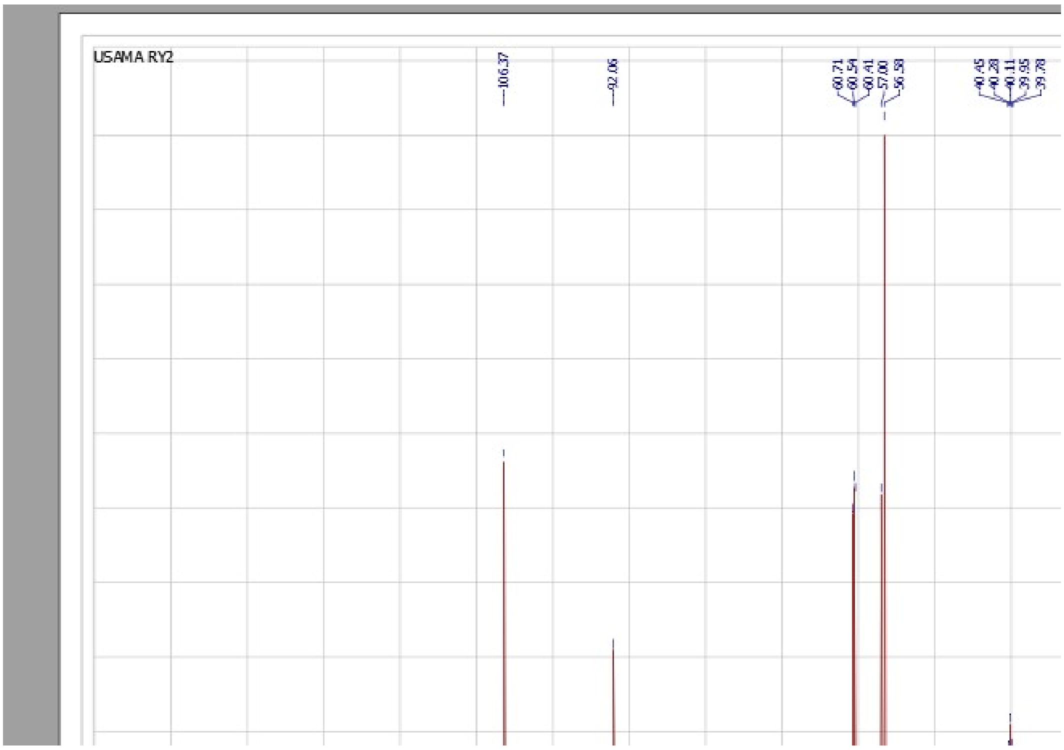

Figure S13: ^{13}C-DEPT NMR spectrum of compound 3 (DMSO-d_6, 500 MHz)

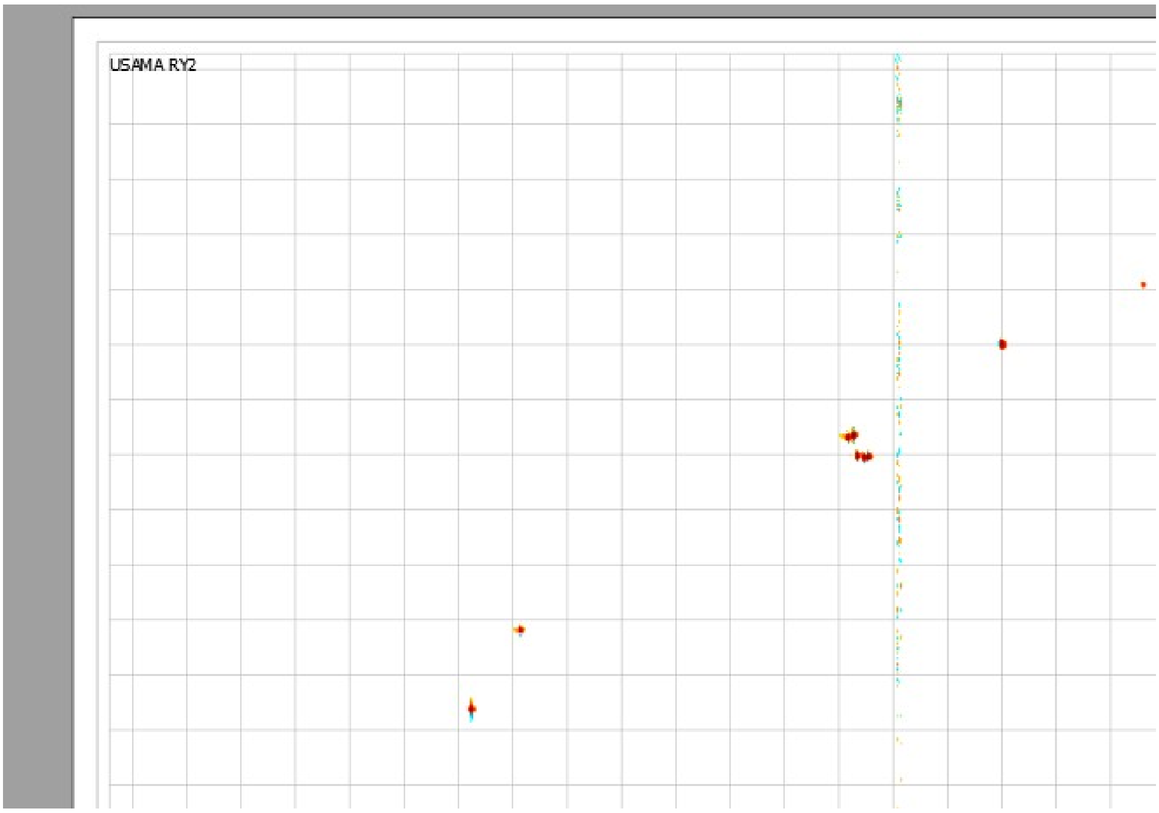

Figure S14: HSQC spectrum of compound 3 (DMSO-d_6, 500 MHz)

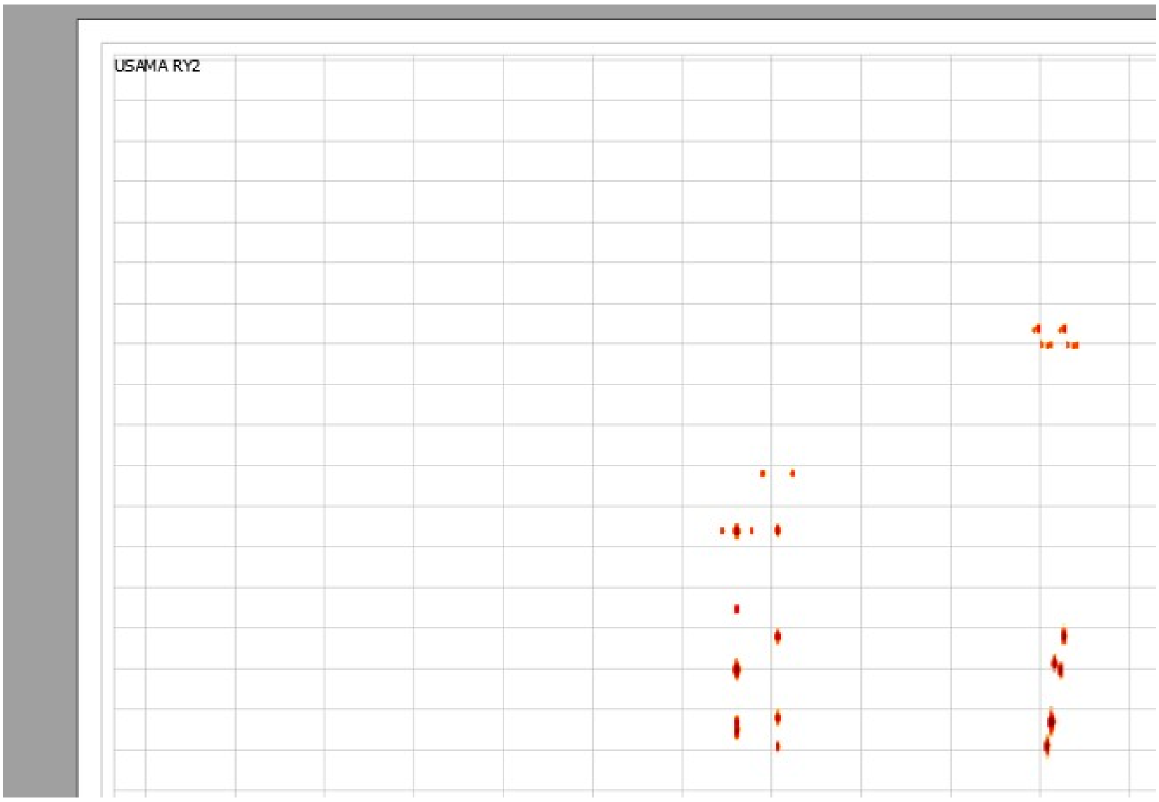

Figure S15: HMBC spectrum of compound 3 (DMSO-d_6, 500 MHz)

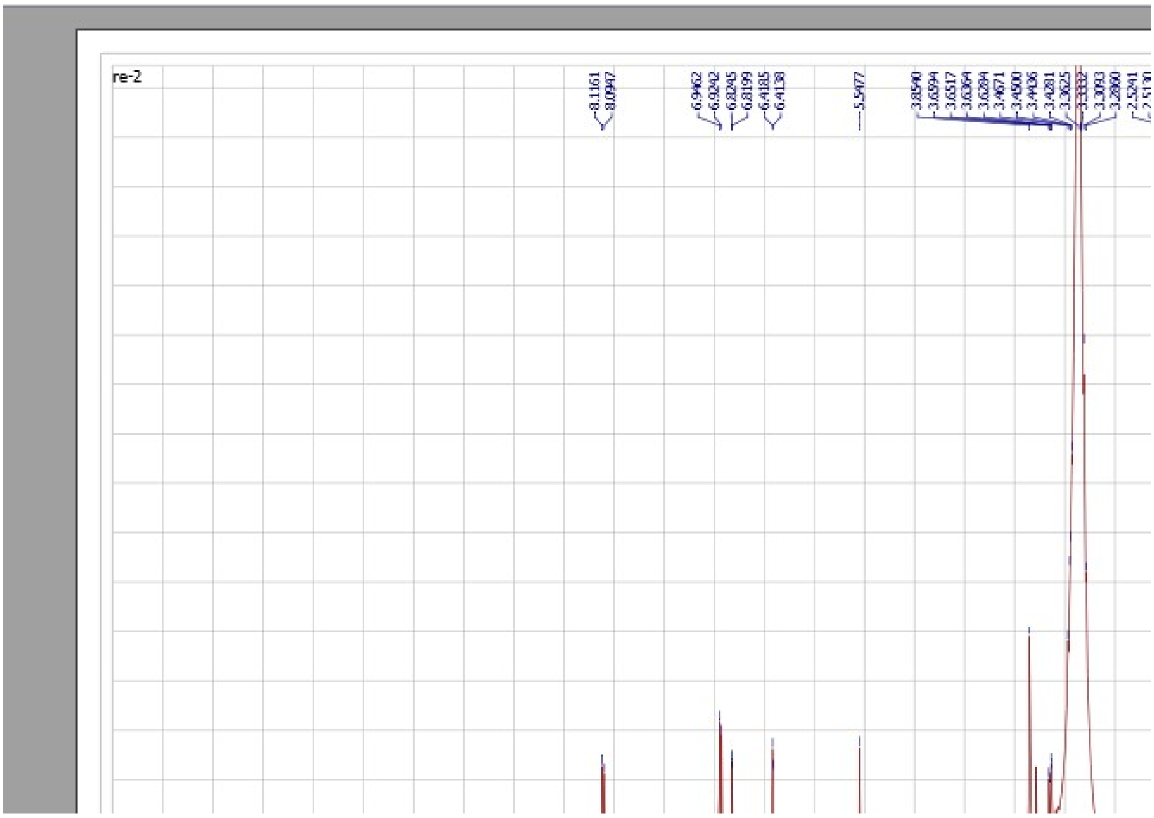

Figure S16: ^{1}H NMR spectrum of compound 4 (DMSO-d_6, 400 MHz)

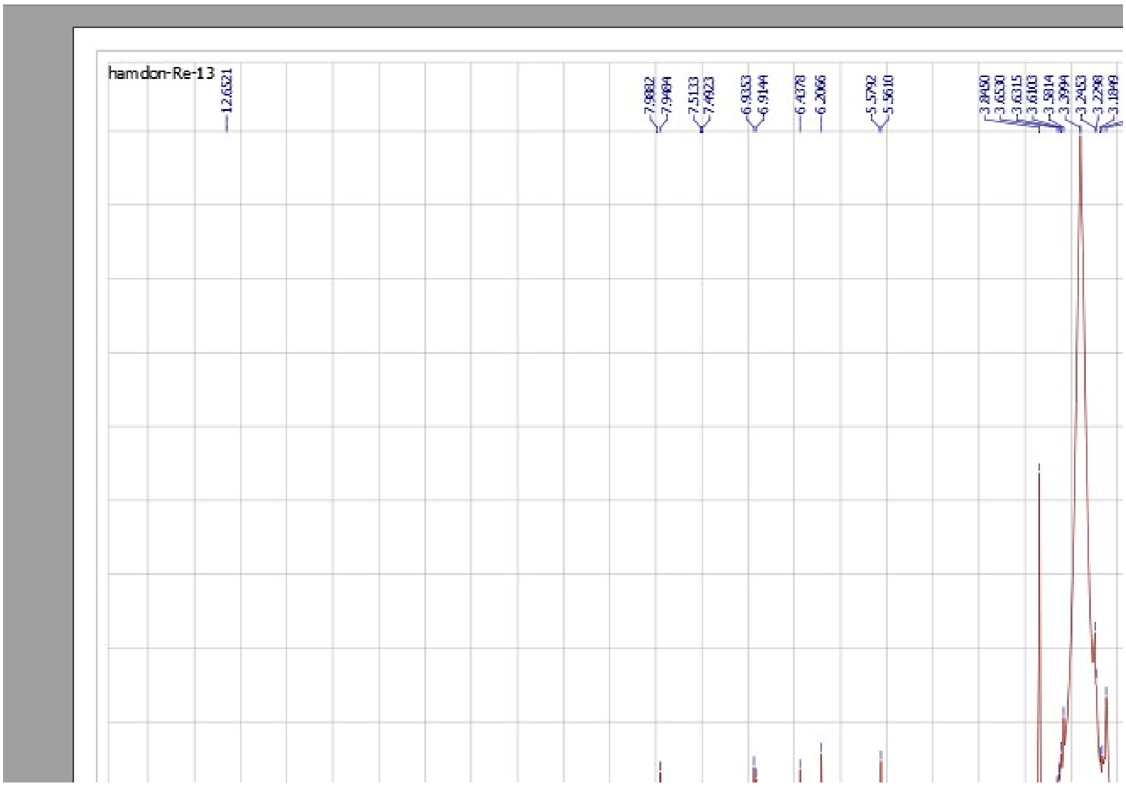

Figure S17: ^{1}H NMR spectrum of compound 5 (DMSO-d_6, 400 MHz)

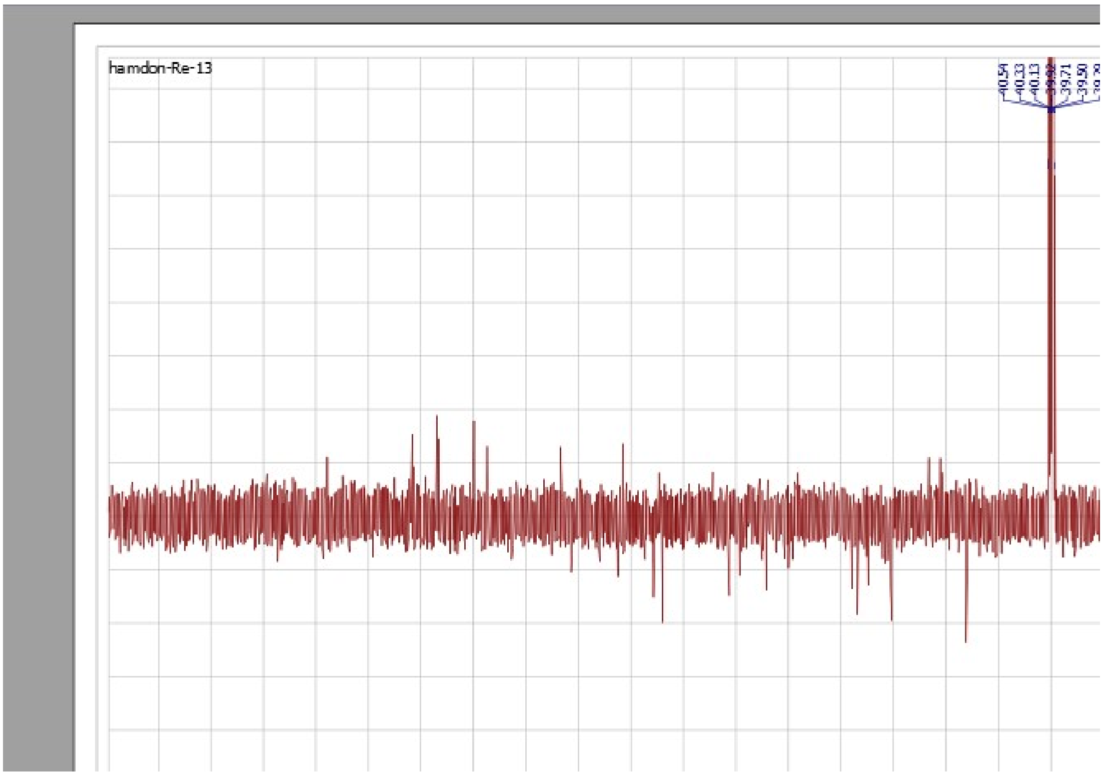

Figure S18: ^{13}C-APT NMR spectrum of compound 5 (DMSO-d_6, 400 MHz)

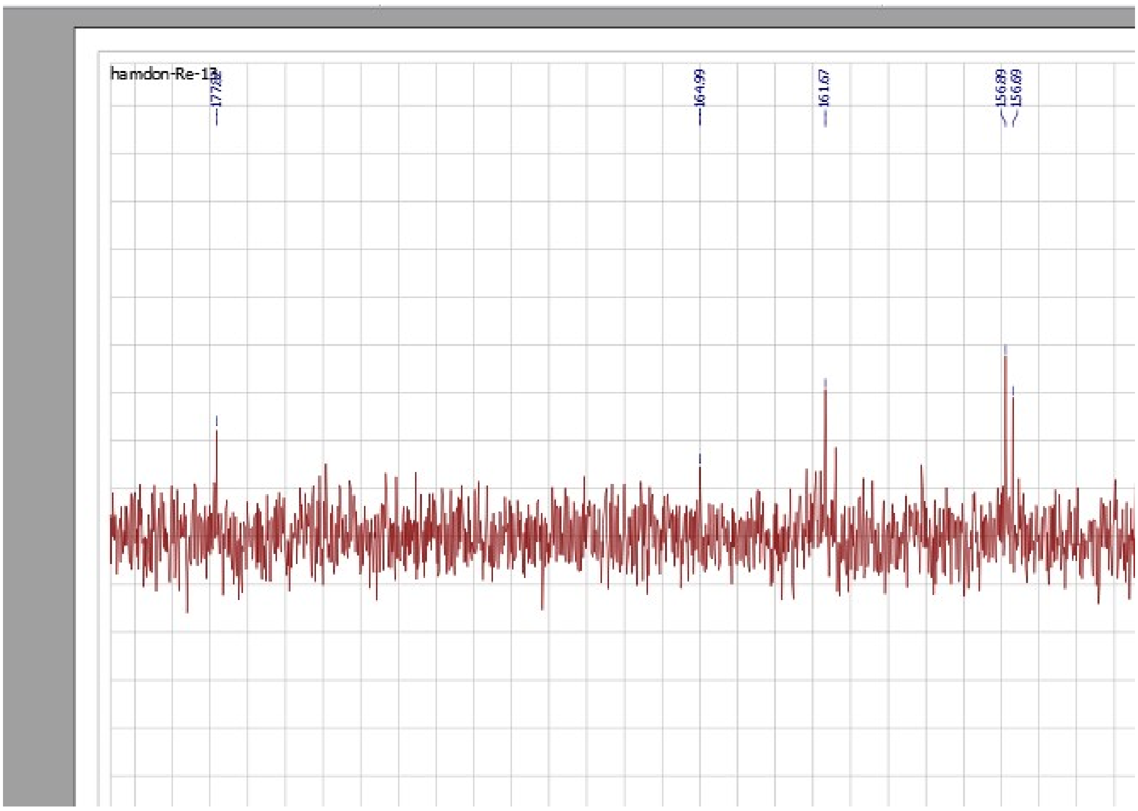

Figure S19: Expanded ^{13}C-APT NMR spectrum of compound 5 (DMSO-d_6, 400 MHz)

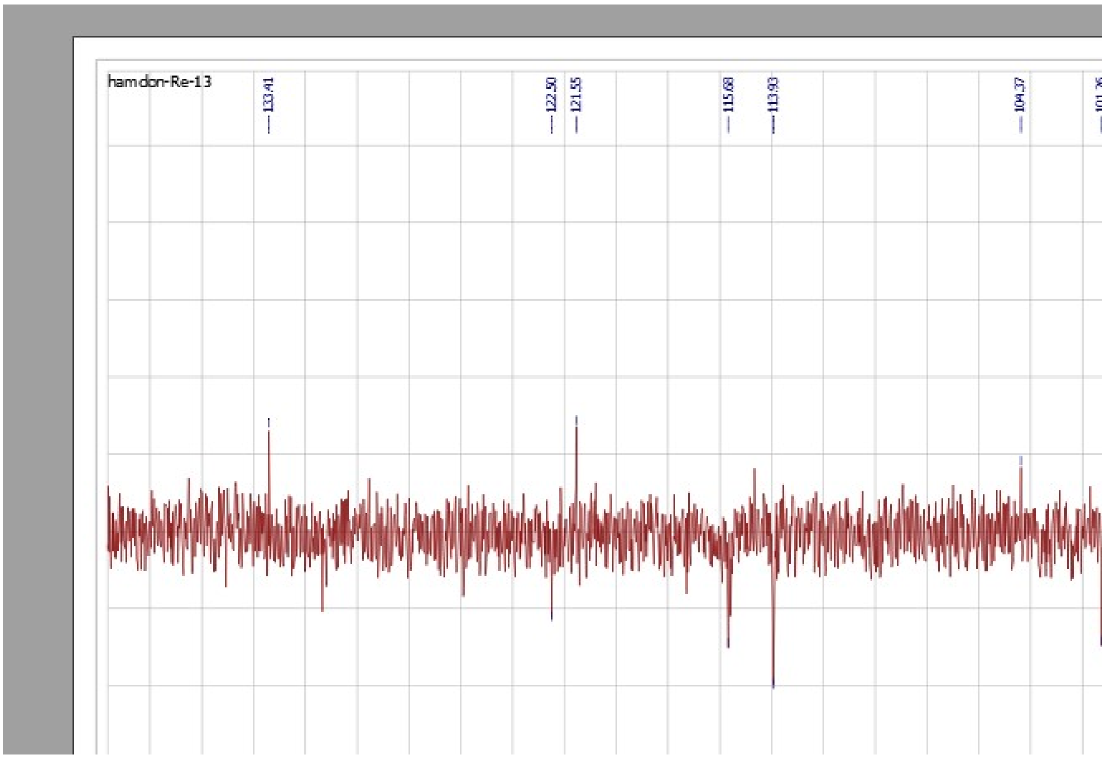

Figure S20: Expanded ^{13}C-APT NMR spectrum of compound 5 (DMSO-d_6, 400 MHz)

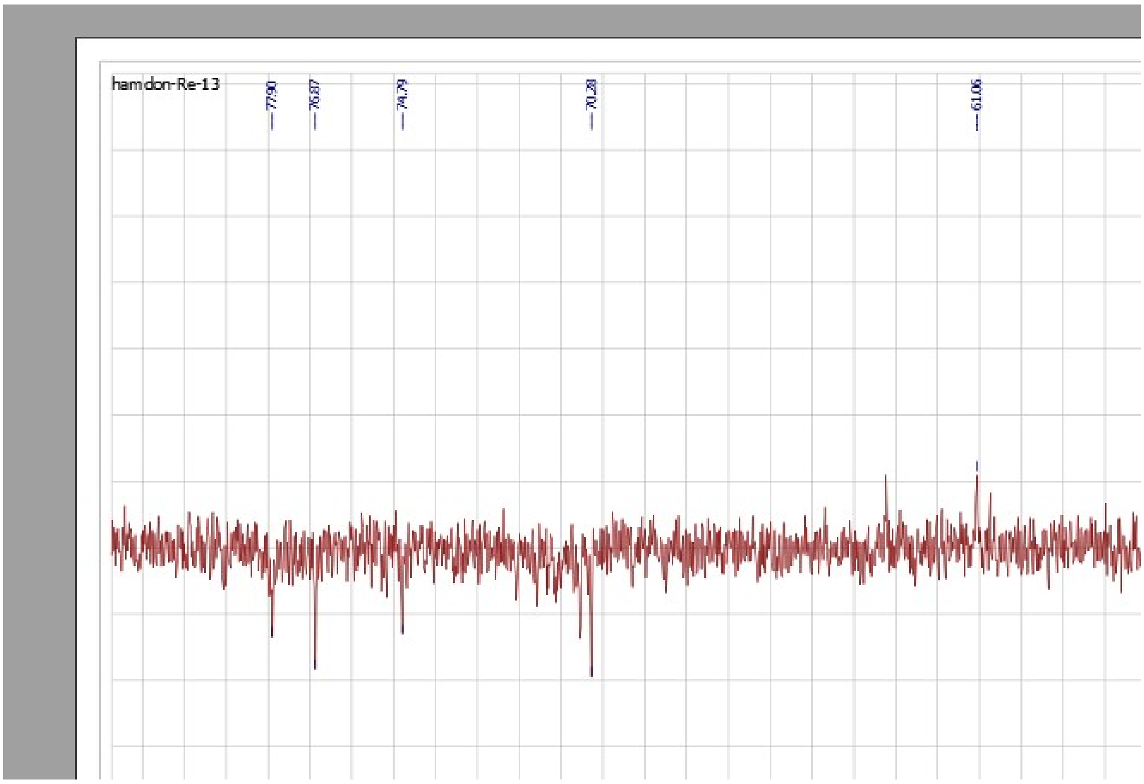

Figure S21: Expanded ^{13}C-APT NMR spectrum of compound 5 (DMSO-d_6, 400 MHz)

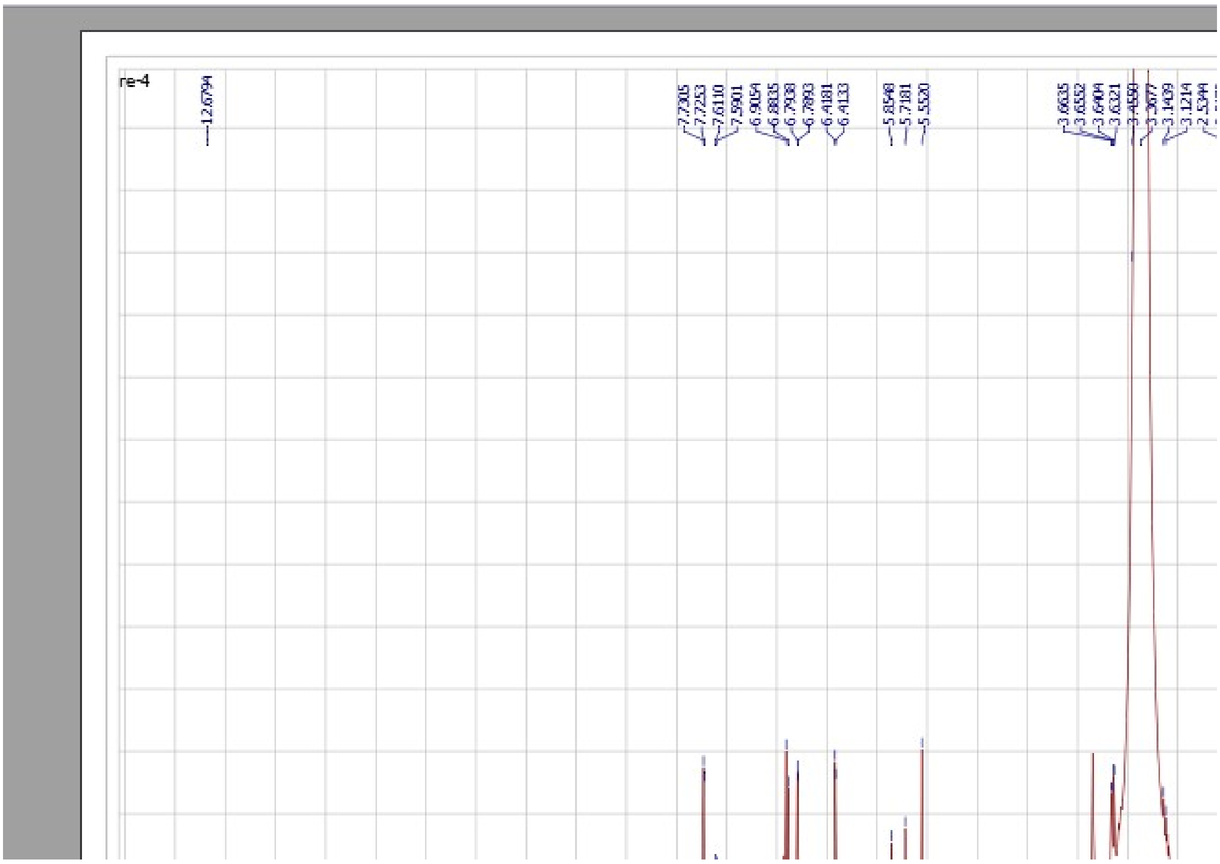

Figure S22: ^{1}H NMR spectrum of compound 6 (DMSO-d_6, 400 MHz)

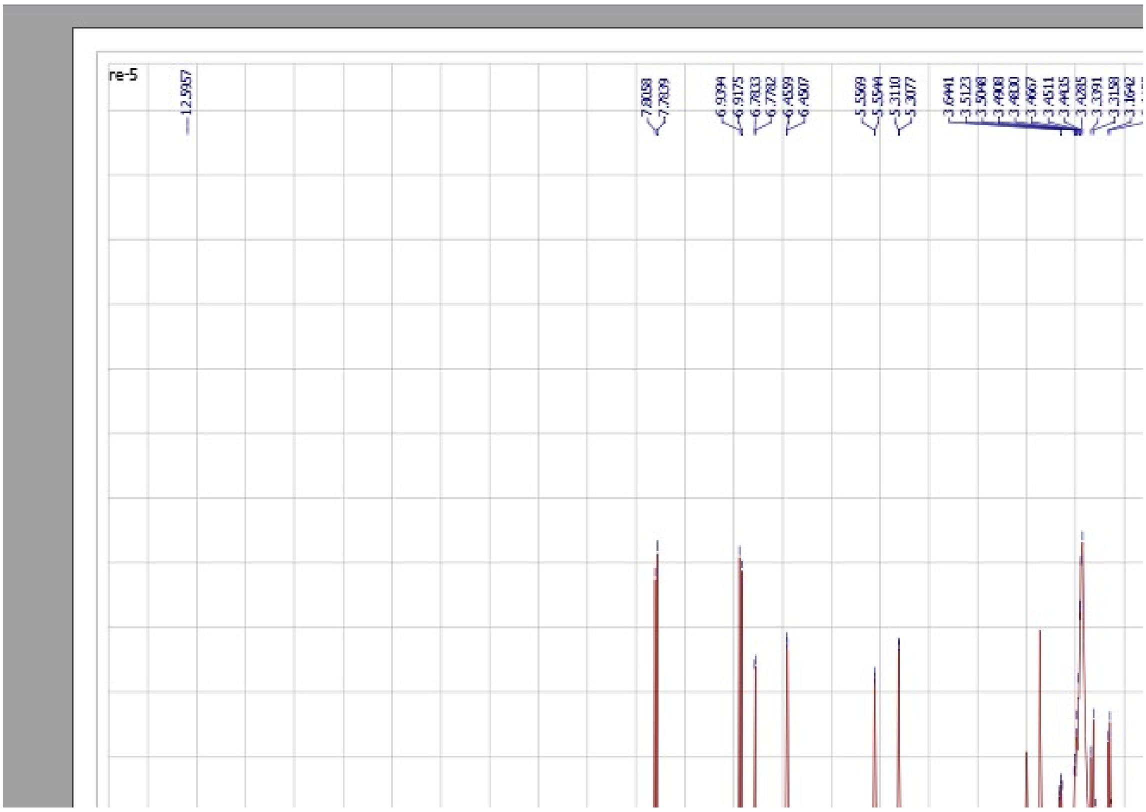

Figure S23: ^{1}H NMR spectrum of compound 7 (DMSO-d_6, 400 MHz)

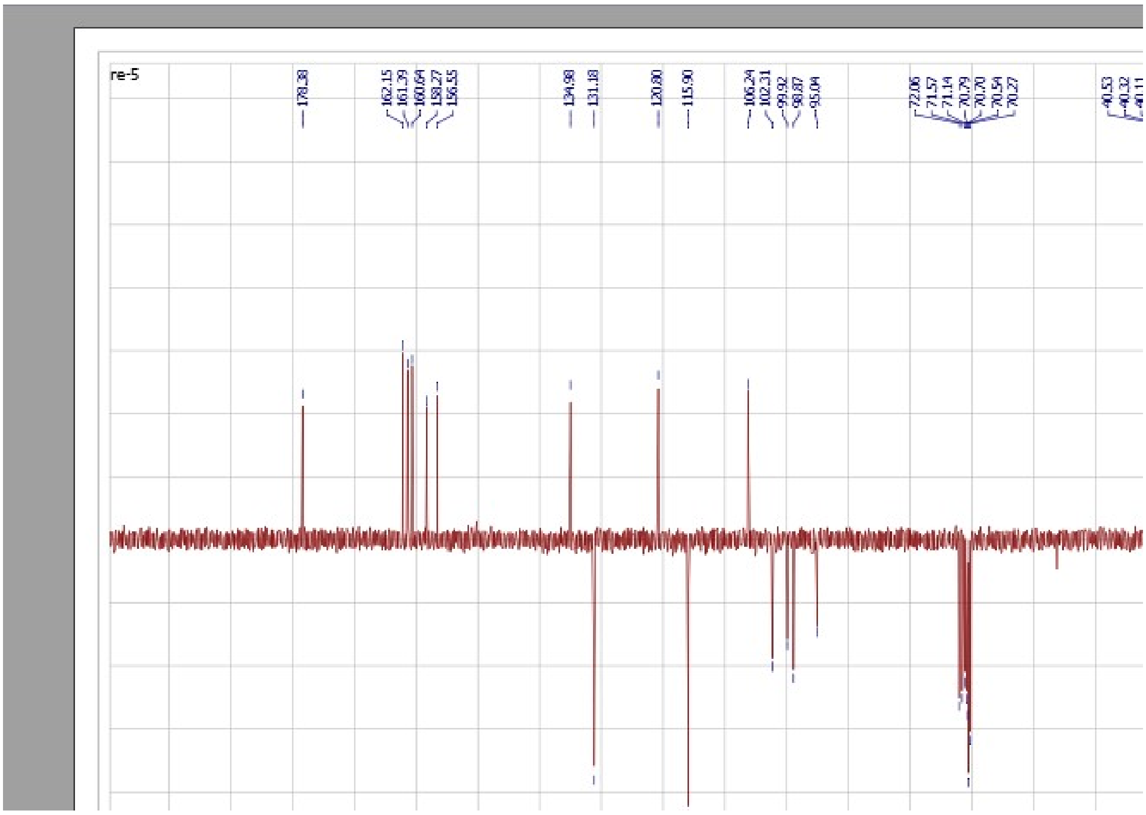

Figure S24: ^{13}C-APT NMR spectrum of compound 7 (DMSO-d_6, 400 MHz)

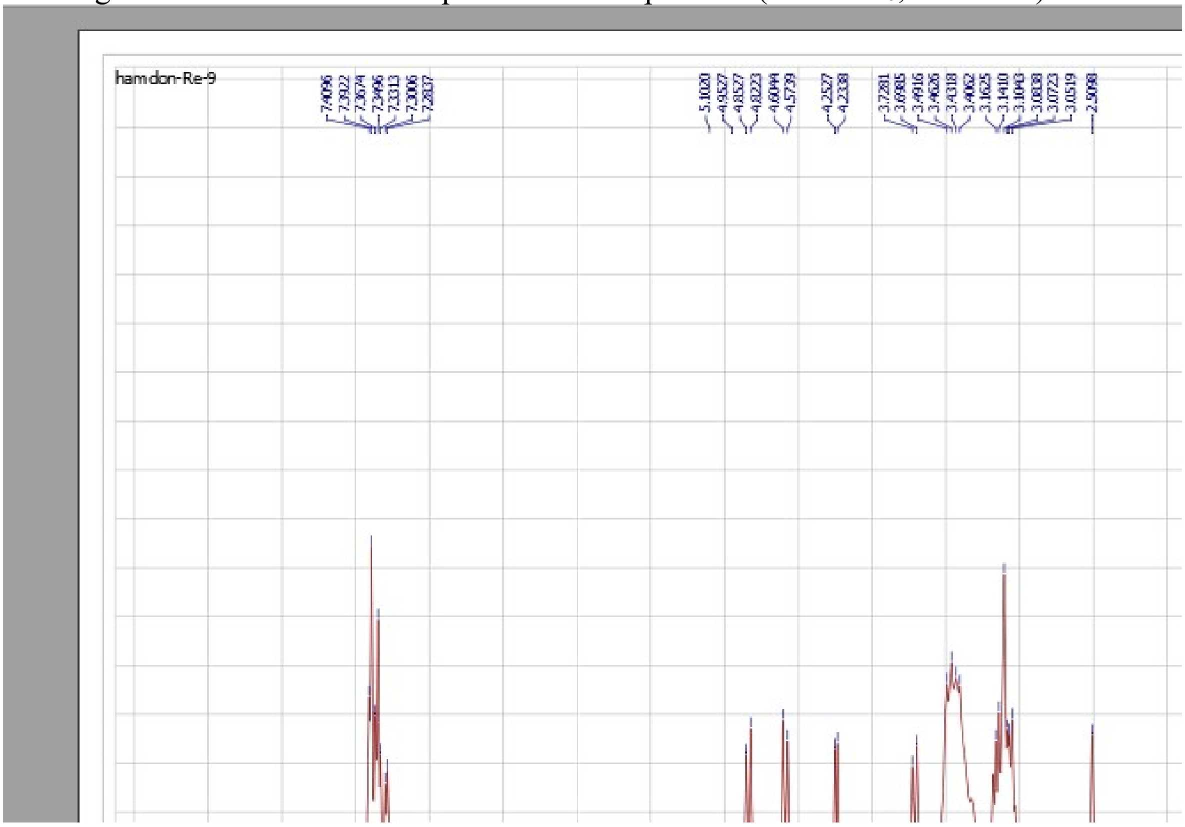

Figure S25: ^{1}H NMR spectrum of compound 8 (DMSO-d_6, 400 MHz)

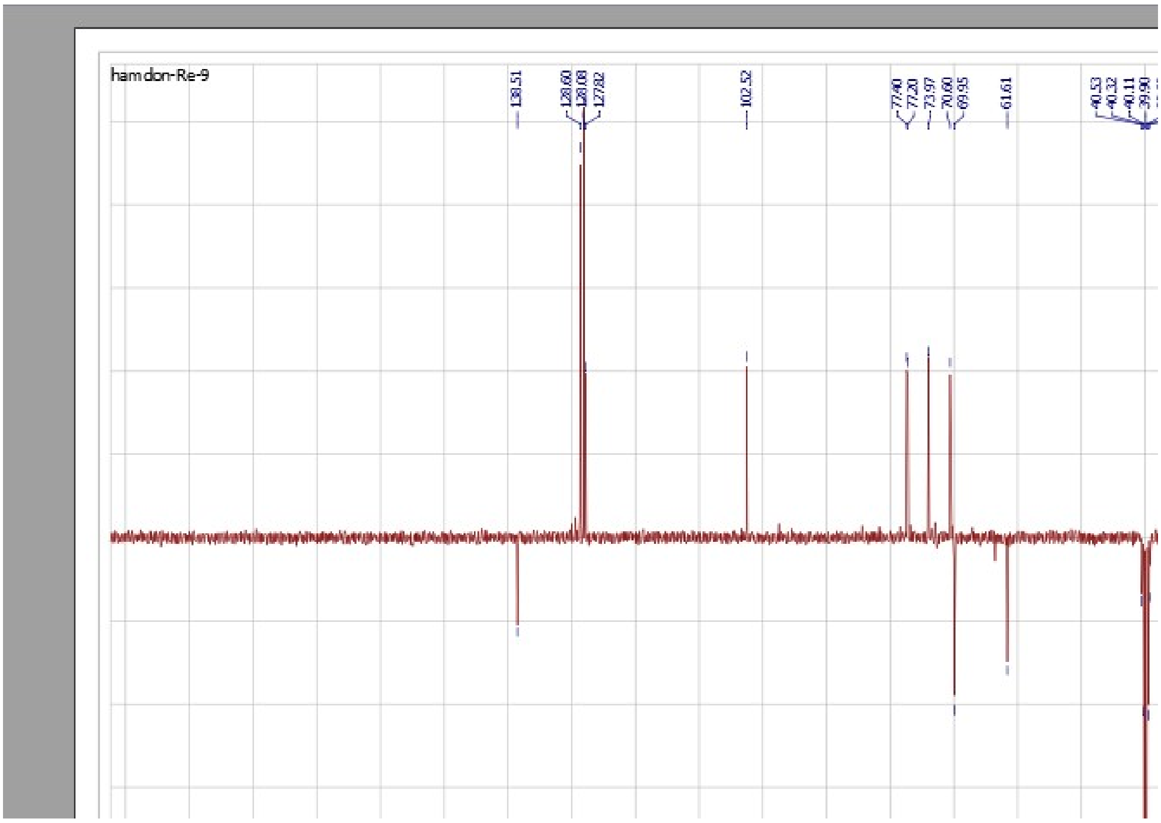

Figure S26: ^{13}C-APT NMR spectrum of compound 8 (DMSO-d_6, 400 MHz)

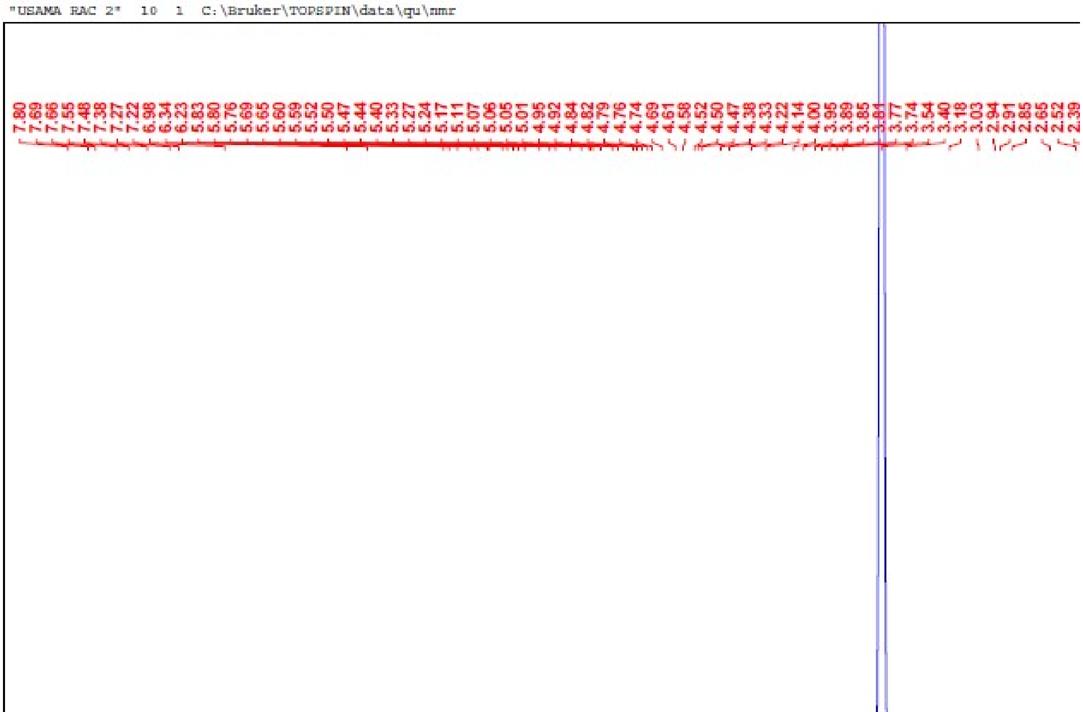

Figure S27: ^{1}H NMR spectrum of compound 9 (DMSO-d_6, 500 MHz)

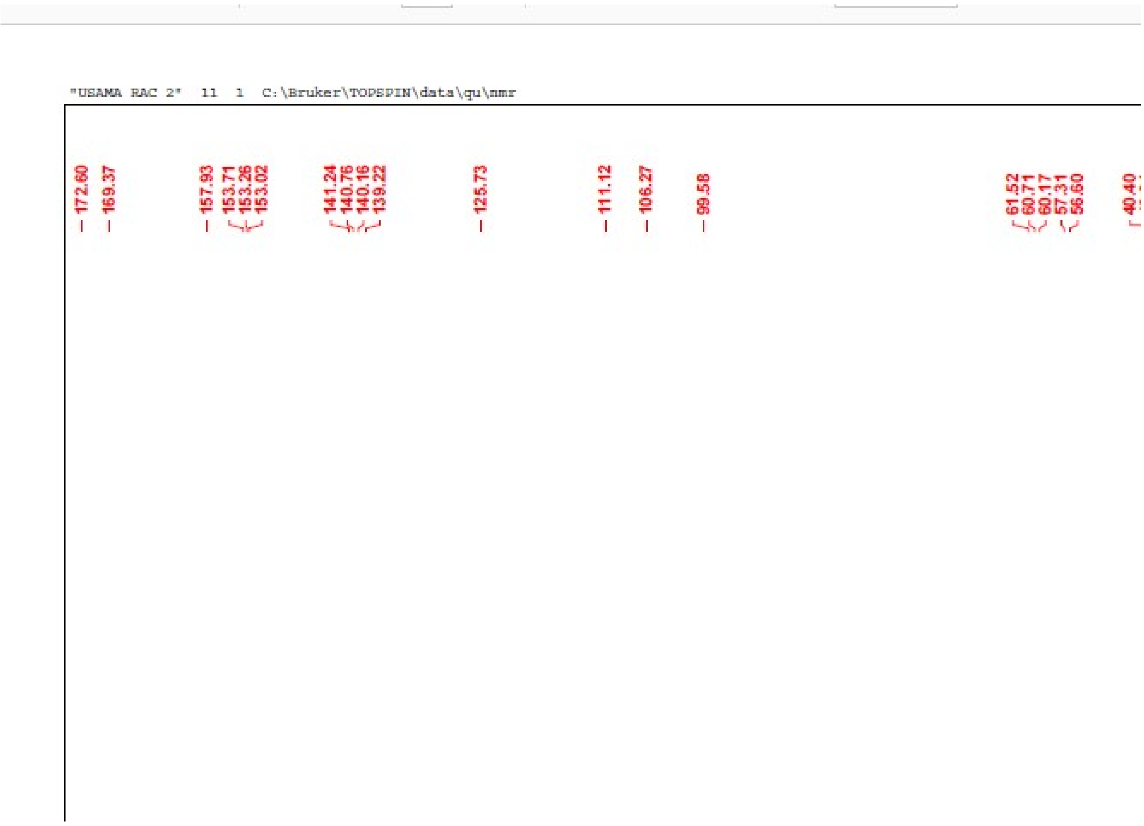

Figure S28: ^{13}C NMR spectrum of compound 9 (DMSO-d_6, 500 MHz)

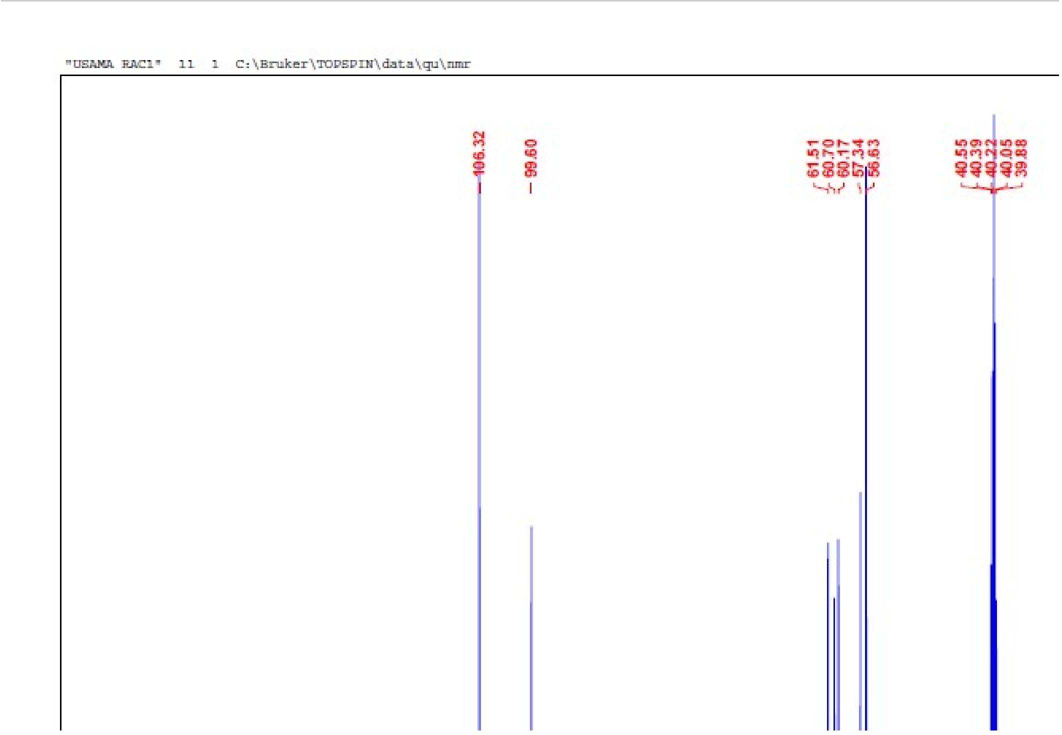

Figure S29: ^{13}C-DEPT NMR spectrum of compound 9 (DMSO-d_6, 500 MHz)

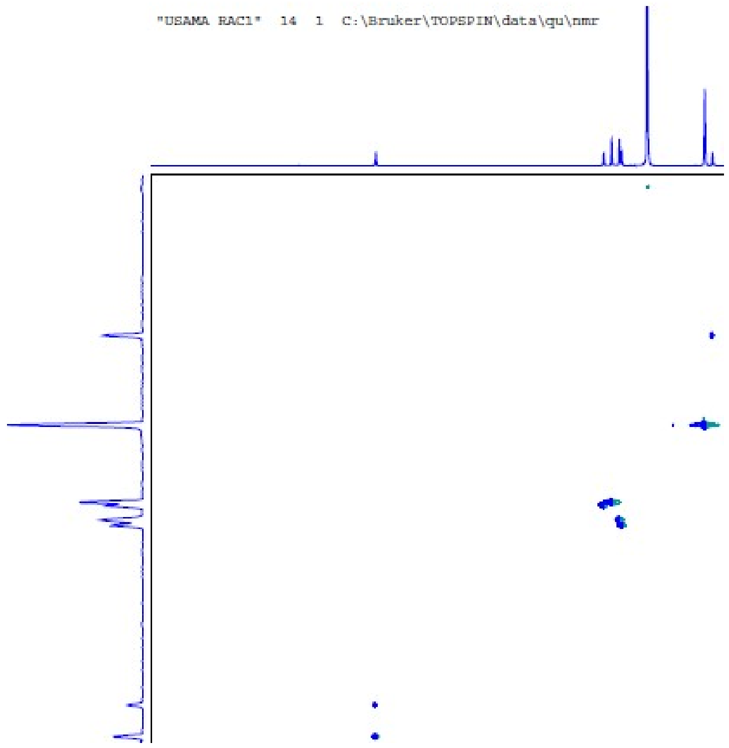

Figure S30: HSQC spectrum of compound 9 (DMSO-d_6, 500 MHz)

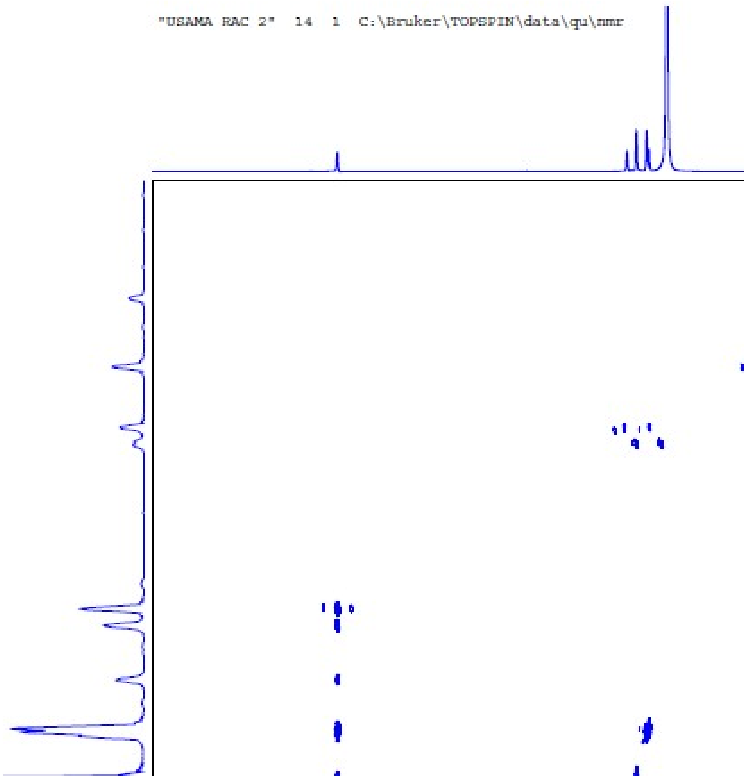

Figure S31: HMBC spectrum of compound 9 (DMSO-d_6, 500 MHz)

Scheme S1: Extraction, fractionation and isolation of chemical constituents of *Rhamnus disperma* roots.

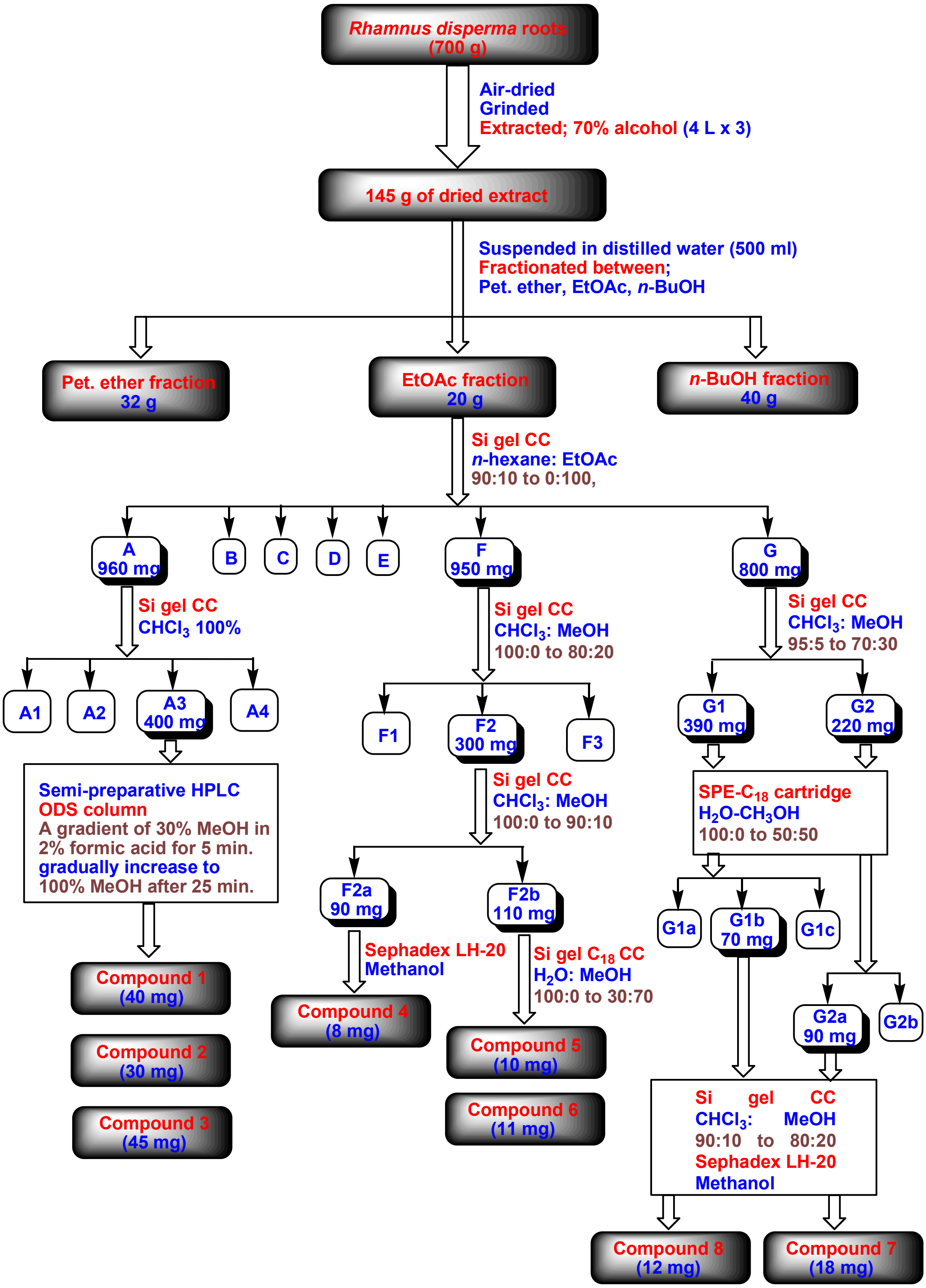

Table S1: NMR spectral data of compound 1 (DMSO-d_6, 500 MHz)

Position	^{1}H	^{13}C	DEPT	HMBC
2	-	156.48	C	-
3	-	138.10	C	-
4	-	178.72	C	-
5	-	152.16	C	-
6	-	132.14	C	-
7		159.13		
8	6.89, s	91.86	CH	C-6, 7, 9, 10
9	-	152.26	C	-
10	-	106.08	C	-
1`	-	120.93	C	-
2`	7.99, d, 7.5	130.67	CH	C-2, 6`, 4`
3`	6.97, d, 7.5	116.19	CH	C-1`, 4`, 5`
4`	-	160.89	C	-
5`	6.97, d, 7.5	116.19	CH	C-1`, 4`, 3`
6`	7.99, d, 7.5	130.67	CH	C-2, 2`, 4`
5-OH	12.63, brs	-	-	-
C-3 OCH_3	3.80, s	60.16	CH_3	C-3
C-6 OCH_3	3.74, s	60.54	CH_3	C-6
C-7 OCH_3	3.92, s	56.96	CH_3	C-7

Table S2: NMR spectral data of compound 2 (DMSO-d_6, 500 MHz)

Position	^{1}H (*J* in Hz)	^{13}C	DEPT	HMBC
2	-	155.80	C	
3	-	138.94	C	
4	-	178.82	C	
5	-	152.02	C	
6	-	132.13	C	
7	-	159.27	C	
8	6.83, s	91.85	CH	C-6, 7, 9, 10
9	-	152.27	C	
10	-	106.13	C	
1`	-	125.35	C	
2`	7.27, s	110.40	CH	C-2, 1`, 3`, 4`, 6`
3`	-	150.96	C	
4`	-	139.47	C	
5`	-	153.49	C	
6`	7.19, s	104.35	CH	C-2, 1`, 2`, 4`, 5`
5-OH	12.49, brs	-	-	C-5, 6, 10
3`-OH	9.70	-	-	
3-OMe	3.80, s	60.37	CH_3	C-3
6-OMe	3.72, s	60.55	CH_3	C-6
7-OMe	3.90, s	56.93	CH_3	C-7
5`-OMe	3.85, s	56.45	CH_3	C-5`
4`-OMe	3.77, s	60.55	CH_3	C-4`

Table S3: NMR spectral data of compound 3 (DMSO-d_6, 500 MHz)

Position	^{1}H (*J* in Hz)	^{13}C	DEPT	HMBC
2	-	155.98	C	
3	-	139.04	C	
4	-	178.84	C	
5	-	152.04	C	
6	-	132.13	C	
7	-	159.30	C	
8	6.93, s	92.06	CH	C-6, 7, 9, 10
9	-	152.30	C	
10	-	106.16	C	
1`	-	125.49	C	
2`	7.39, s	106.39	CH	C-2, 1`, 3`, 4`, 6`
3`	-	153.25	C	
4`	-	140.49	C	
5`	-	153.25	C	
6`	7.39, s	106.39	CH	C-2, 1`, 2`, 4`, 5`
5-OH	12.51, brs	-	-	C-5, 6, 10
3-OMe	3.84, s	60.41	CH_3	C-3
6-OMe	3.73, s	60.54	CH_3	C-6
7-OMe	3.92, s	57.00	CH_3	C-7
3`, 5`-OMe	3.88, s	56.98	CH_3	C-5`
4`-OMe	3.77, s	60.71	CH_3	C-4`
